# Granulocyte colony‐stimulating factor induced T‐cell hyporesponsiveness via modulation of CD177^+^S100A^hi^ neutrophils in unexplained recurrent pregnancy loss

**DOI:** 10.1002/ctm2.70508

**Published:** 2025-10-16

**Authors:** Ping‐Fen Li, Xue Zhang, Peng‐Sheng Zheng

**Affiliations:** ^1^ Department of Reproductive Medicine The First Affiliated Hospital of Xi'an Jiaotong University Xi'an Shaanxi People's Republic of China; ^2^ Department of Reproductive Medicine The Second Affiliated Hospital of Xi'an Jiaotong University Xi'an Shaanxi People's Republic of China; ^3^ Xi'an Peng‐Sheng Reproductive Medicine Clinic Xi'an Shaanxi People's Republic of China

**Keywords:** G‐CSF, PMN‐MDSCs, scRNA‐seq, scTCR‐seq, URPL

## Abstract

**Background:**

Numerous studies have demonstrated the promising efficacy of granulocyte colony‐stimulating factor (G‐CSF) in the treatment of couples with unexplained recurrent pregnancy loss (URPL) during early pregnancy. While neutrophils are recognised as the main effectors mediating immunoregulation, their G‐CSF‐mobilised phenotype and mechanisms regulating maternal–fetal immunity remain unclear.

**Methods:**

Single‐cell RNA sequencing (scRNA‐seq) and single‐cell T‐cell receptor sequencing (scTCR‐seq) were conducted to uncover the immune reconstitution dynamics of peripheral blood under G‐CSF stimulation. Integrative analysis of transcriptomic‐proteomic profiles with functional validation revealed a unique immunomodulatory neutrophil population. Further, we used spatial transcriptomics, flow cytometry and immunohistochemistry to explore the spatial distribution characteristics of this population at the maternal–fetal interface, and validated its therapeutic efficacy in animal models.

**Results:**

G‐CSF‐mobilised peripheral blood (G‐PB) displayed immune hyporesponsiveness. Unique neutrophils expressing high levels of CD177 and the S100A gene family expanded substantially in response to G‐CSF. These neutrophils exhibited a comparatively immature morphology and impaired T‐cell responses via contact‐dependent arginase 1 release, as well as upregulation of T‐cell immune checkpoints. A reduction of CD177^+^S100A^hi^ neutrophils was observed in both peripheral blood and decidua of URPL patients relative to healthy pregnant women. Functional validation in abortion‐prone murine models confirmed that exogenous supplementation of G‐CSF or adoptive transfer of CD177^+^S100A^hi^ neutrophils could successfully improve the pregnancy outcomes.

**Conclusion:**

G‐CSF played a crucial regulatory role in improving pregnancy outcomes by selectively expanding CD177⁺S100A^hi^ neutrophils with polymorphonuclear myeloid‐derived suppressor cells (PMN‐MDSCs) properties, providing a solid theoretical foundation for the treatment of patients with URPL using G‐CSF.

**Key points:**

- G‐CSF induces peripheral blood immune hyporesponsiveness in patients with UPRL.
- First to characterize G‐CSF mobilised CD177^+^S100A^hi^ neutrophils displayed PMN‐MDSCs properties.
- Both CD177^+^S100A^hi^‐LDNs and CD177^+^S100A^hi^‐NDNs induce T cell hyporesponsiveness through coordinated activation of the ARG1/L‐Arg metabolic axis and synergistic upregulation of immune checkpoint molecules.
- CD177^+^S100A^hi^ neutrophils are diminished in the peripheral blood and decidua of patients with UPRL.

## Graphical abstract

G‐CSF‐mobilised CD177⁺S100A^hi^ neutrophils exhibit PMN‐MDSC‐like properties and are significantly reduced in peripheral blood and decidual tissues of URPL patients. These immunosuppressive neutrophil subsets induced T‐cell hyporesponsiveness through coordinated activation of the ARG1/L‐Arg metabolic axis and synergistic upregulation of immune checkpoint molecules, thereby maintaining immune tolerance during pregnancy

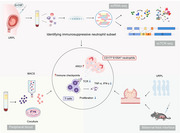

## 1 INTRODUCTION

Embryos carrying half the paternal hereditary information are protected from attack by the maternal immune system, accompanied by implantation during normal pregnancy. This process primarily relies on the complicated and delicate coordination of multiple cells at the maternal–fetal interface.^1^, ^2^ Disruption of the reciprocal tolerance balance causes a series of adverse pregnancy outcomes, such as recurrent pregnancy loss (RPL), defined as two or more spontaneous losses.^3^, ^4^ Epidemiological data indicate a baseline incidence of RPL in 1%–5% of reproductive‐aged women across European and North American populations. Notably, approximately 40%–50% of RPL cases have no identifiable cause, categorised as unexplained RPL (URPL).^5^, ^6^

Despite lacking a conclusive aetiology, alloimmune factors have been proposed to be involved in the occurrence of the URPL. Recent studies have increasingly regarded granulocyte colony‐stimulating factor (G‐CSF) as a chemical mediator actively participating in multiple reproductive processes, including endometrial receptivity, placental metabolism, trophoblast development as well as ovulation (Figure S1).^7^, ^8^, ^9^ Importantly, researchers observed that G‐CSF levels were downregulated in URPL patients, suggesting that the absence of G‐CSF may be a crucial factor in the occurrence of URPL.^10^, ^11^ Meanwhile, Scarpellini et al. revealed that G‐CSF effectively improved the pregnancy outcomes of couples with URPL, especially those with normal embryonic karyotypes.^12^ Analogous findings were obtained in a retrospective trial conducted in 2013,^13^ and a recent meta‐analysis reconfirmed the positive effects of G‐CSF in the treatment of couples with RPL, consistent with observations in murine models.^10^, ^14^ These all suggest that G‐CSF appears to be a promising treatment option for couples with URPL.

Communications of G‐CSF with cells at the maternal–fetal interface have been studied in an attempt to explain the potential immunomodulatory functions, including trophoblast cells, macrophages, dendritic cells, and so forth.^10^, ^15^ Recently, the view that G‐CSF upregulates circulating myeloid‐derived suppressor cells (MDSCs), especially polymorphonuclear myeloid‐derived suppressor cells (PMN‐MDSCs), to regulate immune homeostasis during pregnancy, has attracted attention^16^, ^17^; however, the specific molecular characteristics of involved functional neutrophils mobilised by G‐CSF, their distribution along the neutrophil differentiation trajectory, and differences from conventional PMN‐MDSCs remain elusive.

In this study, we characterise the reconstitution dynamics of peripheral blood (PB) upon G‐CSF stimulation in patients with URPL. G‐CSF‐mobilised peripheral blood (G‐PB) displayed immune hyporesponsiveness, manifested as decreased T‐cell proliferation, reduced production of inflammatory factors, and impaired T‐cell receptor (TCR) repertoire. Importantly, we identified an expanded PMN‐MDSC‐like neutrophil subpopulation immunophenotyped as CD177^+^S100A^hi^ neutrophils in G‐PB, which was diminished in PB and decidua of URPL patients. Multi‐omics demonstrated that the expansion of CD177^+^S100A^hi^ neutrophils and their suppression of T cells may be crucial mechanisms for the effective treatment of patients with URPL using G‐CSF.

## 2 MATERIALS AND METHODS

### 2.1 Patient samples

This research was conducted in accordance with the Declaration of Helsinki and approved by the Institutional Review Board of the Ethics Committee of the School of Medicine, Xi'an Jiaotong University (XJTU1AF2024LSYY‐430). PB samples were systematically collected from three distinct cohorts: patients with URPL, patients with URPL receiving G‐CSF treatment, and age‐matched healthy pregnant controls (HCs). The inclusion criteria and clinical characteristics of the samples are described in the supplementary materials. Additionally, 10 age‐matched healthy non‐pregnant female volunteer donors were included in this study for the isolation of pan‐T cells.

First‐trimester decidual tissue samples were collected from the above three cohorts. These samples were obtained from individuals undergoing elective surgical termination of pregnancy for non‐medical indications at the above hospital department. All samples were obtained from the Department of Obstetrics and Gynaecology at the First Affiliated Hospital of Xi'an Jiaotong University, and processed within 1–2 h of collection.

### 2.2 scRNA‐seq

PB samples were collected before and after G‐CSF treatment from six early pregnant women with a history of URPL. After red blood cell lysis, the cells were centrifuged at 500 *× g* for 5 min and resuspended in phosphate‐buffered saline (PBS). An automatic cell counter (Countstar) was applied to count the cells, and the cell viability of each sample was evaluated to be above 95%. Subsequently, the single‐cell suspension was loaded into the microfluidic chip to construct the scRNA‐seq library suitable for Illumina sequencing according to the protocols of the GEXSCOPE Single Cell RNA Library Kit (Singleron Biotec). Sequencing was performed on the Illumina HiSeq X10 platform using a 150 bp paired‐end reading strategy. Statistical power for scRNA‐seq data was evaluated through differential gene expression (DE) and expression quantitative trait loci analyses using the scPower.^18^

### 2.3 scTCR sequencing

As previously described,^19^ full‐length TCR fragments were enriched from amplified cDNA to construct scTCR sequencing libraries suitable for Illumina sequencing in accordance with the instructions of the GEXSCOPE Single Cell Immuno‐TCR Kit (Singleron Biotec). The library sequencing strategy was the same as that used for single‐cell sequencing. Each TCR α‐chain–TCR β‐chain pair was considered a clonotype, and the clones were grouped accordingly.

### 2.4 Cell isolation

Peripheral blood mononuclear cells (PBMCs) and normal density neutrophils (NDNs) were harvested from patients with URPL treated with G‐CSF using a Neutrophil Isolation Kit (TBD Sciences). According to the manufacturer's protocols, neutrophils were purified by CD15 microbeads (Miltenyi Biotec). CD177^+^S100A^hi^‐low‐density neutrophils (LDNs) and CD177^+^S100A^hi^‐NDNs were sorted from the PBMCs and NDNs fractions in G‐PB by surface staining with FITC‐conjugated anti‐human CD177 antibody and co‐incubation with anti‐FITC microbeads (Miltenyi Biotec). CD2^+^CD3^+^CD5^+^CD7^+^‐pan‐T cells were collected from the PBMCs of healthy donors using the ‘Pan‐T‐cell Isolation kit’ (Miltenyi Biotec) and Lymphoprep (Stemcell). The purity of all sorted cells was assessed using a BD FACS Aria II (BD Biosciences).

### 2.5 Cell morphological analysis

Sorted CD177^+^S100A^hi^‐LDNs and CD177^+^S100A^hi^‐NDNs were stained with Diff‐Quick stain after centrifugation following the manufacturer's instructions (TBD Sciences). Images were captured using a microscope (Nikon Eclipse 80i) equipped with a 100× oil immersion objective.

### 2.6 T‐cell proliferation assays

Untouched sorted pan‐T cells were stained with 1 µM CellTrace Far Red (Invitrogen) and resuspended in RPMI 1640 (Gibco) containing 10% fetal bovine serum (HyClone), 1% superglutamine, and 1% penicillin/streptomycin. Stained pan‐T cells (2 × 10^4^) were co‐cultured with or without sorted CD177^+^S100A^hi^‐LDNs or CD177^+^S100A^hi^‐NDNs at ratios of 5:1, 1:1 and 1:5 in a round‐bottom 96‐well plate with CD3/CD28 activator (Stemcell). After 96 h, the Far Red staining intensity was measured using flow cytometry to assess T‐cell proliferation. In some experiments, the following inhibitors were added: 2 mM *N*‐acetylcysteine (NAC, Sigma‐Aldrich), 200 µg/mL L‐arginine (L‐Arg, Sigma‐Aldrich), and 200 U/mL catalase (CAT, Sigma‐Aldrich). Subsequently, a 0.4 µm Transwell (Costar, Corning) was applied to detect T‐cell proliferation without direct contact with neutrophils. T cells were placed in the lower chamber of the Transwell plate, while neutrophils were added to the upper chamber.

### 2.7 Flow cytometry analysis

Cells were treated with Fc receptor blocking solution (BD Biosciences) before staining with surface antibodies. A total of 1 × 10^5^ cells were suspended in 50 µL stain buffer (BD Biosciences), and the following fluorescein‐conjugated antibodies were added and stained for 30 min at 4°C in the dark: CD3, CD4, CD8, CD45 (BD Biosciences), CD15 and CD177 (BioLegend). Intracellular staining of ARG1 (BD Biosciences) and S100A8/A9 (BD Biosciences) was conducted using the Cytofix/Cytoperm Fixation/Permeabilisation Kit (BD Biosciences). For reactive oxygen species (ROS) analysis, a DHE fluorescent probe was used to detect ROS levels in CD177^+^S100A^hi^ neutrophils (Huaxingbio). Apoptosis levels were measured using the PE Annexin V Apoptosis Detection Kit I (BD Biosciences), as described previously.^20^ Cytokines were detected by multiplex microsphere flow cytometry immunofluorescence luminescence following the instructions in the kit (Raisecare Biotech). All data were processed using FlowJo 10.0 software (FlowJo).

### 2.8 Proteomic analysis

T cells cultured alone and exposed to CD177^+^S100A^hi^‐LDNs were collected by flow cytometric sorting. The cell pellet was mixed with lysis buffer and lysed at 95°C for 10 min, followed by NanoDrop protein quantification (NanoDrop One, Thermo), and the peptide was purified and reconstituted before mass spectrometry analysis and library generation. Spectronaut built‐in Pulsar (version 18) was used to build a spectral library with DIA data and matched to the UniProt Homo sapiens database (protein number: 26 766).

### 2.9 Immunohistochemistry and immunofluorescence

The following procedures were performed for immunohistochemical staining of paraffin sections as we described previously^21^: deparaffinised, hydration, heat‐mediated antigen retrieval, BSA blocking, primary antibody incubation in the dark at 4°C overnight, incubation with secondary antibody the next day, DAB colour development, and haematoxylin counterstaining.

Immunofluorescence was performed using the Treble‐Fluorescence Immunohistochemistry Mouse/Rabbit Kit (ImmunoWay Biotechnology) following the manufacturer's instructions. For each slide, 10 representative and non‐overlapping views were randomly selected under a 20× microscope to count the positive cells. The average number of stained cells in 10 fields was regarded as the number of positive cells and recorded as cells/high‐power field. The antibodies used are listed in Table S6.

### 2.10 Enzyme‐linked immunosorbent assay (ELISA) assay

TNF‐α, IFN‐γ and L‐Arg in the supernatant harvested from the T cells/ CD177^+^S100A^hi^ neutrophils co‐culture system were measured using the corresponding ELISA kits (MultiSciences Biotech; Coab Biotech).

### 2.11 Spatial visualisation

Spatial locations and expression of CD177 and S100A family genes at the human maternal–fetal interface were visualised using the ‘Explore’ module of the CROST database (https://ngdc.cncb.ac.cn/crost, VISDP000138).^22^ Corresponding spatial mapping for the mouse maternal–fetal interface was generated through an interactive data portal (https://db.cngb.org/stomics/mpsta/).^23^

### 2.12 Experimental animals and grouping

The mice used in the in vivo study (6‐week‐old CBA/J female mice, BALB/c male mice, and DBA/2 male mice) were purchased from Beijing SPF Biotech Co. Ltd. The mice were reared in a pathogen‐free environment with constant temperature and humidity, a 12‐h light/dark cycle, and free access to water and food for 2 weeks to acclimate. An abortion‐prone (AP) model was implemented in female CBA/J mice mated with DBA/2 mice.^24^ CBA/J female mice were naturally mated with BALB/c male mice to establish the normal pregnancy (NP) model. Mated female mice were examined for vaginal plugs at 7:00 the next day. The presence of a vaginal plug was designated as pregnancy day 0.5.

Pregnant mice received intraperitoneal injections of recombinant G‐CSF or saline control from gestational day (gd) 4.5 to 13.5, or intravenous administrations of isolated mouse‐derived CD177⁺S100A^hi^ neutrophils at gd4.5 and gd6.5. AP mice were randomly divided into three groups: AP‐saline group, AP‐G‐CSF group (50 µg/kg),^10^ and AP‐CD177^+^S100A^hi^‐neutrophils group. NP mice were administered saline as a pregnancy control. Pregnant mice were euthanased at gd10.5 or gd14.5. Uteri were harvested for molecular biological analyses, and the numbers of absorbed embryos and viable embryos were quantified and photographically documented.

### 2.13 Mouse‐derived CD177^+^S100A^hi^ neutrophils adoptive transfer

Neutrophils were isolated from the PB of G‐CSF‐treated female CBA/J mice using a murine‐specific neutrophil isolation kit. Subsequently, CD177⁺S100A^hi^ neutrophils were isolated by fluorescence‐activated cell sorting following sequential staining with Ly‐6G, CD177 (BD Biosciences) and S100A9 (Abclonal). The sorted cells were resuspended in 200 µL of sterile PBS and intravenously injected into pregnant CBA/J mice (2 × 10^6^ cells/mouse) via the tail vein at gd4.5 and gd6.5.^25^, ^26^

### 2.14 Statistical analyses

Differences in variables were calculated using paired or unpaired Student's *t*‐test and Wilcoxon rank‐sum test (two groups) and one‐way ANOVA with Tukey's post hoc test (multiple groups) using GraphPad Prism 7 (GraphPad Software Inc.). Statistical significance was set at *p* < 0.05, as specified in the figure legends.

## 3 RESULTS

### 3.1 G‐CSF mediates immune hyporesponsiveness in PB

**FIGURE 1:**
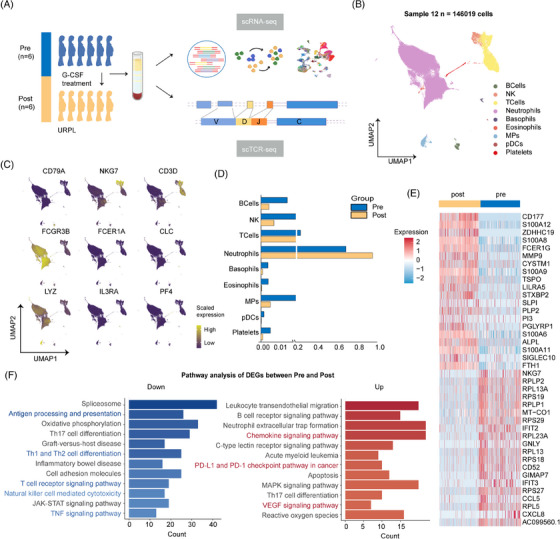
PB transcriptome landscape after G‐CSF treatment in pregnant women with URPL. (A) Workflow illustration of the scRNA‐seq experimental design. (B) UMAP of 146 019 single cells from six donors before and after G‐CSF treatment. Coloured by cell clusters. (C) UMAP of canonical marker genes specifically distinguished each lineage. (D) Proportion of each cluster in G‐CSF mobilised PB compared with unstimulated PB. Colours represent group, pre indicates samples before G‐CSF treatment and post represents samples after G‐CSF treatment. (E) Heatmap showing expression of top 20 DEGs before and after G‐CSF stimulation. (F) Enriched pathways of downregulated (blue) and upregulated (red) genes before and after G‐CSF treatment. PB, peripheral blood; URPL, unexplained recurrent pregnancy loss; MPs, mononuclear phagocyte cells; pDCs, plasmacytoid dendritic cells; scRNA‐seq, single‐cell RNA‐seq; UMAP, uniform manifold approximation and projection; DEGs, differentially expressed genes.

We utilised PB samples from six early pregnant women with a clinical diagnosis of URPL before and after G‐CSF administration for single‐cell transcriptome analysis on a microfluidic chip (Figure 1A). The basic clinical and laboratory characteristics of the recruited pregnant patients are described in Table S1. After rigorous quality filtering, 146 019 high‐quality cells were retained and integrated into a comparable and unbatched data library (Figure S2A; Table S3). Of these, 69 731 cells (47.8%) were derived from samples taken before treatment, whereas the remaining 76 288 cells (52.2%) were from samples taken after G‐CSF administration, with robust statistical power (both expression probability and DE power >0.8).^18^ Based on reported gene signatures, we preliminarily annotated the composition of cell clusters in PB, namely B cells, natural killer (NK) cells, T cells, neutrophils, basophils, eosinophils, mononuclear phagocyte (MP) cells, plasmacytoid dendritic (pDC) cells and platelets (Figure 1B,C; Figure S2B). The distribution of the 12 PB samples in these clusters was highly reproducible, indicating a strong degree of fit (Figure S2C,D).

G‐CSF administration triggered dramatic neutrophil expansion concurrent with T/NK cluster contraction, molecularly mirrored by upregulation of granulocyte markers (CD177, S100A8/12, MMP9) and downregulation of lymphocyte differentiation genes (CD3D, NKG7, TRAC, ZAP70) (Figure 1D,E). Differentially expressed genes (DEGs) before and after G‐CSF treatment were extracted for enrichment analysis, which showed that downregulated genes were significantly enriched in immune‐related pathways, such as T‐cell differentiation, antigen processing and presentation and TNF signalling pathways (Figure 1F). Gene Set Enrichment Analysis corroborated this immunoregulatory feature (Figure S2E), suggesting that G‐CSF, as an important intercellular communication mediator, promotes the transformation of the peripheral immune system to a low‐responsive phenotype.^8^, ^9^, ^27^

### 3.2 Heterogeneous neutrophil differentiation trajectories in response to G‐CSF stimulation

**FIGURE 2:**
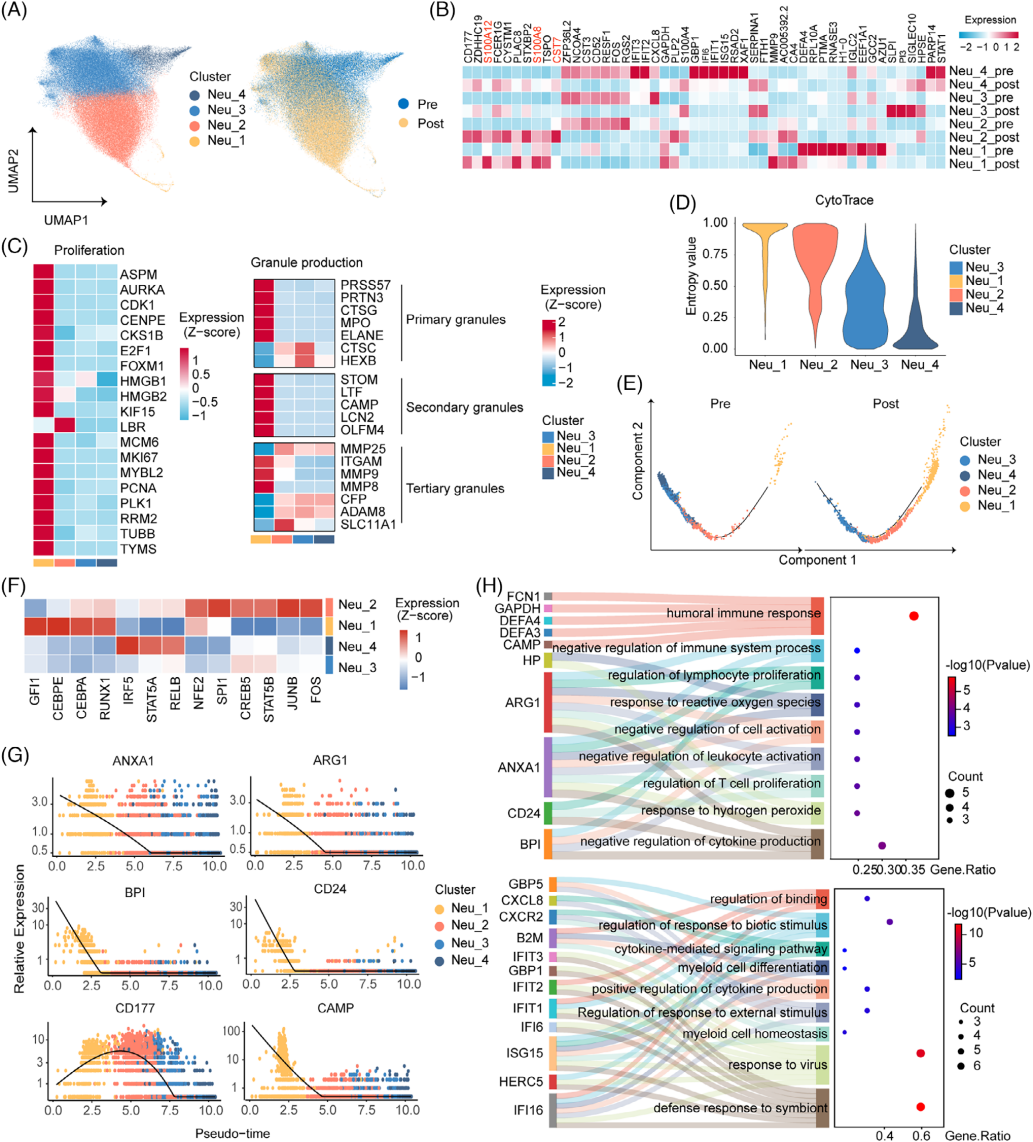
Profile of neutrophils differentiation and developmental trajectory under G‐CSF administration. (A) UMAP visualisation of subclusters in neutrophils. Coloured according to cell states (left) and sample stage (right). (B) Heatmap displaying average expression of DEGs among four neutrophil subclusters before and after G‐CSF administration. Pre indicates samples before G‐CSF treatment and post represents samples after G‐CSF treatment. (C) Heatmap of the proliferation‐ (left) and granule‐related genes (right) expression among four neutrophil subclusters. Data are presented as a *z*‐score of gene expression. (D) Violin plot showing entropy/cyto‐trace value of four neutrophil subclusters. The *x* and *y*‐axis, respectively, indicate cell clusters and corresponding entropy/cyto‐trace values. (E) Prediction tree of neutrophil differentiation trajectories mapped by Monocle upon G‐CSF treatment. (F) Heatmap displaying *z*‐score of scaled expression of TFs among four neutrophil subclusters. The *y*‐axis represents clusters, and the *x*‐axis lists TFs. (G) Top six genes that are differentially expressed along with pseudo‐time. The *x*‐axis from left to right represents the pseudo‐time from small to large. The *y*‐axis indicates gene expression level. Coloured by subclusters. (H) Enriched pathways of DEGs (state1, top; state2, bottom) along with pseudo‐time. UMAP, uniform manifold approximation and projection; DEGs, differentially expressed genes; TFs, transcription factors.

Neutrophils were the main target cells of G‐CSF, with a short lifespan, marked plasticity and significant heterogeneity.^28^, ^29^ In our study, unbiased clustering analysis identified four distinct neutrophil states in the PB, which were represented by individual subclusters (Figure 2A,B; Figure S3A,B). Neu‐1, the least abundant in PB, remarkably enriched the expression of proliferation‐ and cycle‐related genes (MKI67, CDK1 and PCNA), as presented by Gene Set Variation Analysis, and it exhibits a preference towards PRTN3, MPO and CTSG, which are involved in primary granule synthesis (Figure 2C; Figure S3C). Additionally, Neu‐1 and Neu‐2 displayed marked upregulation of immunoregulatory genes (S100A12, MMP9, CD177), indicating that they may be involved in the regulation of the immune microenvironment. Neu‐3 lacked a distinct signature, suggesting that it may be in a transitional state. And Neu‐4 exhibited a specific expression profile reminiscent of terminal granulocyte subclusters, characterised by a high abundance of IFIT1, IFIT2, IFIT3, IFI6, ISG15 and HERC5 (Figure 2B; Figure S3A).^30^, ^31^

To systematically characterise the lineage associations among the four neutrophil subclusters, we ranked and coloured the cells along possible developmental trajectories in pseudo‐time. Neu‐1, closely associated with cell proliferation, displayed the strongest stemness. It was located at the root of the trajectory and showed a directional flow toward the remaining subclusters (Figure 2D,E; Figure S3E). Furthermore, Neu‐1 highly expressed RUNX1 and several integral transcription factors (TFs) crucial for early neutrophil differentiation, such as CEBPA, CEBPE and GFI1 (Figure 2F).^28^, ^32^, ^33^ And TFs RUNX1 and CEBPA were more active in Neu‐2 than in the other two subclusters (Figure 2F). Expression profiling of surface protein‐encoding genes further highlighted the immature phenotype shared by Neu‐1 and Neu‐2 (Figure S3D). These transcriptional signatures and pseudotemporal analyses collectively establish Neu‐1 and Neu‐2 as the initial stage of PB neutrophil development, with Neu‐3 functioning as a transient intermediary bridging Neu‐1/Neu‐2 and the terminally differentiated Neu‐4 population.

Surprisingly, these subclusters at various stages of differentiation were not specific for critical genes involved in phagocytosis and chemotaxis (Figure S3F). Therefore, we extracted DEGs along the trajectory toward mature neutrophils. As differentiation proceeded, the levels of arginase 1 (ARG1), ANXA1, CST7, DEFA3 and CAMP were gradually downregulated, whereas those of the interferon‐stimulated genes were upregulated. Functional enrichment analysis further demonstrated that early immature neutrophils (Neu‐1/Neu‐2) exhibited immunosuppressive properties, with functional transition to antiviral defence and immune activation emerging during maturation (Figure 2G,H; Figure S3G).

### 3.3 CD177^+^S100A^hi^ neutrophils have an PMN‐MDSCs‐like phenotype

**FIGURE 3:**
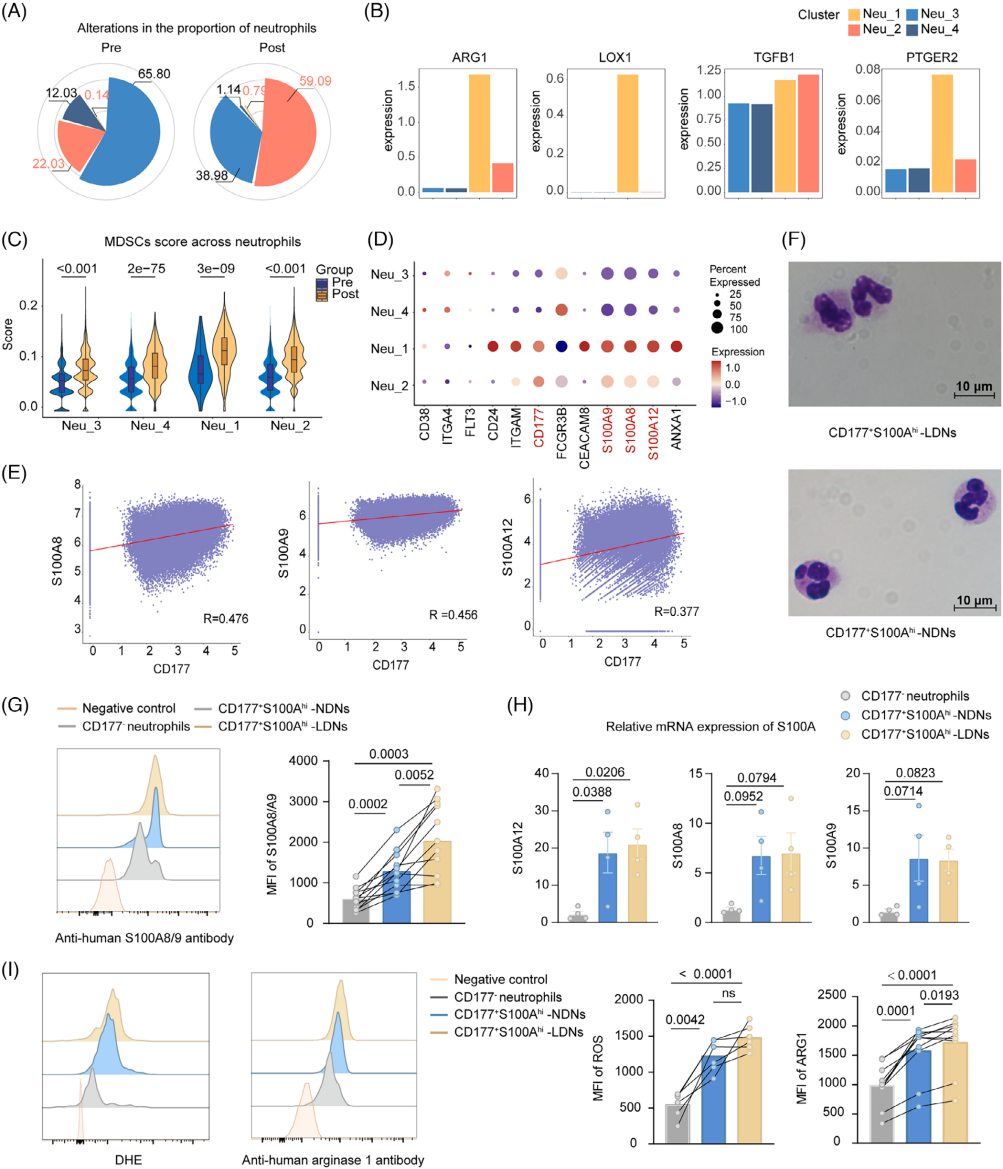
CD177^+^S100A^hi^ neutrophils exhibit a PMN‐MDSCs‐like phenotype. (A) Alternation of four neutrophil subcluster proportions responding to G‐CSF stimulation. (B) Boxplot showing average expression of factors mediating the immunosuppressive properties of MDSCs across four neutrophil subclusters. (C) Violin plots showing MDSC scores of four neutrophil subclusters before and after G‐CSF stimulation. (D) Dotplot showing average expression of candidate surface marker genes among four neutrophil subclusters. (E) Pearson correlation analysis of the expression levels of CD177 and S100A family gene (S100A8, S100A9 and S100A12). (F) Morphological characterization of CD177^+^S100A^hi^ neutrophils isolated from the mononuclear cell fraction and the ‘normal density’ neutrophils after G‐CSF administration. Scale bar: 10 µm. (G) Flow cytometry of S100A8/A9 expression in sorted G‐CSF mobilised CD177^+^S100A^hi^‐LDNs, CD177^+^S100A^hi^‐NDNs and remaining CD177^−^ neutrophils (*n* = 12/each group). (H) Relative mRNA expression of S100A12 (left), S100A8 (middle) and S100A9 (right) in sorted G‐CSF mobilised CD177^+^S100A^hi^‐LDNs, CD177^+^S100A^hi^‐NDNs and remaining CD177^−^ neutrophils (*n* = 4/each group). Data are represented as mean ± SEM. (I) Flow cytometry of ROS (left, *n* = 6/each group) and ARG1 (right, *n* = 10/each group) expression in sorted G‐CSF mobilised CD177^+^S100A^hi^‐LDNs, CD177^+^S100A^hi^‐NDNs and remaining CD177^−^ neutrophils. *p*‐values are calculated using the one‐way ANOVA with Tukey's post hoc test. PMN‐MDSCs, polymorphonuclear myeloid‐derived suppressor cells; MDSCs, myeloid‐derived suppressor cells; MFI, mean fluorescence intensity; LDNs, low‐density neutrophils; NDNs, normal density neutrophils; ARG1, arginase 1; ROS, reactive oxygen species.

Considering that the proportion of neutrophils was substantially augmented in G‐PB, we calculated the relative proportions within the neutrophil lineage to deeply dissect the dynamics of G‐CSF‐driven neutrophil differentiation. Higher percentages of Neu‐1 and Neu‐2 were observed after G‐CSF administration, with the most substantial increase in Neu‐2 (Figure 3A). Notably, several upregulated genes, such as S100A8, S100A12, MMP9 and OLR1 in MDSCs, also accumulated at high levels in Neu‐1 and Neu‐2 (Figure S3A).^34^, ^35^ Gene set scoring showed that these two clusters exhibited higher MDSC scores (Figure S4A). Factors mediating the immunosuppressive properties of MDSCs were also upregulated (Figure 3B).^36^, ^37^ And this tendency was more evident after the G‐CSF stimulation (Figure 3C; Figure S4B,C), suggesting immature neutrophils, represented by Neu‐1 and Neu‐2, accumulated in G‐PB and may possess immunosuppressive potential similar to that of MDSCs.

On this basis, we identified several surface protein‐encoding genes to isolate immunosuppressive neutrophils. CD177 emerged as a principal candidate marker, showing predominant enrichment and exclusive expression in Neu‐1 and Neu‐2 versus other neutrophil populations (Figure 3D). Transcriptomic analysis revealed co‐expression between CD177 levels and S100A family genes, such as S100A8, S100A9 and S100A12, prompting targeted isolation (Figure 3E). Through magnetic bead selection, we sorted CD177^+^ cells from the PBMCs and the NDNs fractions of G‐PB (purity >99.0%, Figure S4D). Consistent with the scRNA‐seq, there was an obvious upregulation of S100A8/A9/A12 in CD177^+^ cells, with concurrent elevation observed at both transcriptional (mRNA) and translational (protein) levels. Therefore, these immunophenotypically distinct subpopulations were denominated as CD177^+^S100A^hi^‐LDNs (PBMCs origin) and CD177^+^S100A^hi^‐NDNs (NDNs fraction), respectively (Figure 3G,H).

Morphologically, CD177^+^S100A^hi^‐LDNs were mainly a mixed population composed of band cells and metamyelocytes, whereas the nuclear morphology of CD177^+^S100A^hi^‐NDNs was more mature and mostly triply segmented (Figure 3F). Moreover, we tested PMN‐MDSC‐associated effector molecules—ROS and ARG1—through flow cytometric analysis. Quantitative analysis revealed significantly elevated ROS/ARG1 expression in CD177⁺S100A^hi^ neutrophils compared to control populations (Figure 3I), confirming their identity as an immunophenotypically distinct subpopulation with immunosuppressive capacity in PB.

### 3.4 Hyporesponsiveness of T and NK lineages under G‐PB conditions

**FIGURE 4:**
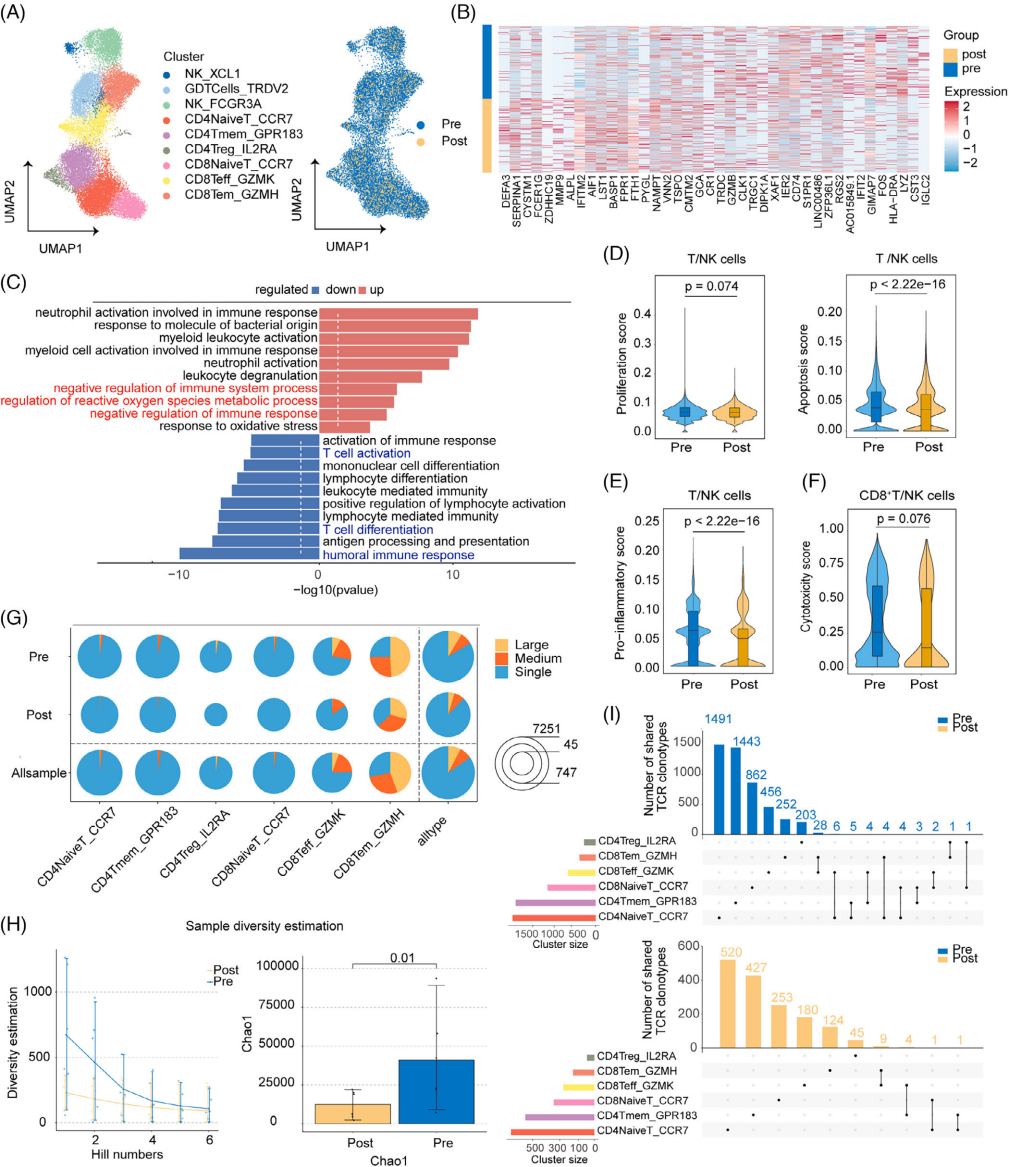
The impacts of G‐CSF on T and NK lineage in PB. (A) UMAP and re‐clustering of T and NK cells. Assigned identities to subclusters based on canonical marker expressions. Coloured by cell states (left) and sample stage (right). (B) Heatmap showing the expression of DEGs of T/NK subclusters between unstimulated PB and G‐PB. (C) Enrichment of GO terms for the upregulated and downregulated DEGs of T cells in G‐PB compared with unstimulated PB. The length of each bar denotes −log 10(*p*‐value). (D) Violin plot of proliferation score (left) and apoptosis score (right) in T/NK cells in unstimulated PB compared with G‐PB. (E) Violin plot of proinflammatory score in T/NK cells before and after G‐CSF treatment. (F) Violin plot of cytotoxicity score in CD8^+^ T/NK cells in unstimulated PB compared with G‐PB. (G) Pie chart of distribution of different clone states and grouped clonotype frequencies in each T‐cell cluster. (H) Clonal diversity performance after G‐CSF administration inferred by Hill (left) and Chao1 algorithm (right). The *y*‐axis represents the clonal diversity of the sample. (I) UpSet plot showing number of shared TCR clonotypes across different clusters before and after G‐CSF treatment. *p*‐values are calculated using the Wilcoxon rank‐sum test. PB, peripheral blood; UMAP, uniform manifold approximation and projection; DEGs, differentially expressed genes; G‐PB, G‐CSF‐mobilised peripheral blood; GO, gene ontology; TCR, T‐cell receptor.

Given the pivotal regulatory role of PMN‐MDSCs in T‐cell suppression, we systematically characterised the immunophenotypic remodelling of T and NK lineages under G‐CSF stimulation. According to canonical cell markers labelling, nine subclusters were acquired: GD T cells (TRDV2^+^), three subtypes of CD4^+^ T clusters, three subtypes of CD8^+^ T clusters, and two subtypes of NK clusters (KLRF1^+^) (Figure 4A; Figure S5A,B). G‐CSF administration induced a marked contraction in total T‐ and NK‐cell populations. Within this lymphopenic landscape, we observed that naive CD4^+^T cells in homeostatic proliferation state showed a relative expansion, whereas CD4⁺ memory T cells (Tmem), CD4⁺ regulatory T cells (Tregs), and CD8⁺ granzyme K‐expressing effector T cells (Teff‐GZMK) increased only slightly (Figure S5C,D). And T and NK cells in G‐PB demonstrated enrichment of immunosuppressive genes such as LST1, CR1 and MAPK14, whereas genes involved in T‐cell activation and differentiation (HLA‐DRA, FOS and CD74) and immunomodulatory molecules (IGHA1, LYZ and IFIT2) were downregulated (Figure 4B). Enrichment analysis of DEGs also confirmed that the negative regulation of the immune response was enhanced after G‐CSF treatment. Notably, enhanced neutrophil and myeloid cell activation in immune responses was observed, suggesting that the crosstalk between T cells and neutrophils was not one‐way. T cells may indirectly modulate neutrophil activity via cytokine‐mediated signalling or intercellular interactions (Figure 4C).^38^, ^39^, ^40^

Gene scoring analysis revealed obvious suppression of proliferative activity in both T‐ and NK‐cell compartments within G‐PB, with CD8⁺ T cells exhibiting the most pronounced reduction. While apoptosis scores unanimously decreased compared with the unstimulated group, preliminarily ruling out apoptotic pathways as a mechanism for G‐CSF‐mediated immunosuppression (Figure 4D; Figure S5E,F). Moreover, there were attenuated inflammatory responses in G‐PB, characterised by diminished proinflammatory signature scores (suppressed expression of TNF‐α, IFN‐γ and IL‐1β), impaired Th1‐polarisation score, and reduced myeloid activation markers (CD68 expression) (Figure 4E; Figure S5G–I). Cytotoxic potential was compromised, as evidenced by depressed cytotoxicity scores in both CD8⁺ T and NK cells (Figure 4F; Figure S5J). These findings collectively established a broad immunosuppressive state in G‐PB.

Complementing transcriptional profiling, we systematically characterised the TCR repertoire. As a unique and stable identifier of T‐cell clones, the TCR sequence was used to further evaluate the T‐cell response.^41^, ^42^ A total of 5969 clonal types were identified: 4651 from the unstimulated group and 1441 from the treatment group (Figure S6A–C). On this basis, we categorised clonal expansion into three groups: large (clone frequency >10), medium (10 ≥clone frequency >1), and single (a single clone). Clonal expansion was visibly absent in G‐PB, with the most notable reduction observed in large ones (Figure 4G; Figure S6D,E). Three patients experienced a complete loss of large‐scale clonal frequency after treatment, which occurred primarily in the CD8^+^ Tem‐GZMH and CD8^+^ Teff‐GZMK subclusters (Table S4). A similar phenomenon occurred with clonal diversity; there was a decrease in the diversity upon G‐CSF stimulation, indicating that G‐CSF may hinder the effective expansion of effector T‐cell clones (Figure 4H; Figure S6F). Inter‐cluster clonal cells were also investigated. Among two or more subclusters, 58 shared TCR clonotypes were observed under homeostasis, whereas only 15 were shared after G‐CSF treatment (Figure 4I). Among these, CD8^+^ Tem‐GZMH and CD8^+^ Teff‐GZMK cells displayed relatively high proportions of inter‐cluster clones. This pattern was consistent across samples; however, the similarity was lost in the treatment group, further emphasising the immunosuppressive state of T cells under G‐CSF stimulation.

### 3.5 G‐CSF mobilised CD177^+^S100A^hi^ neutrophils suppress T‐cell responses via contact‐dependent ARG1 release

**FIGURE 5:**
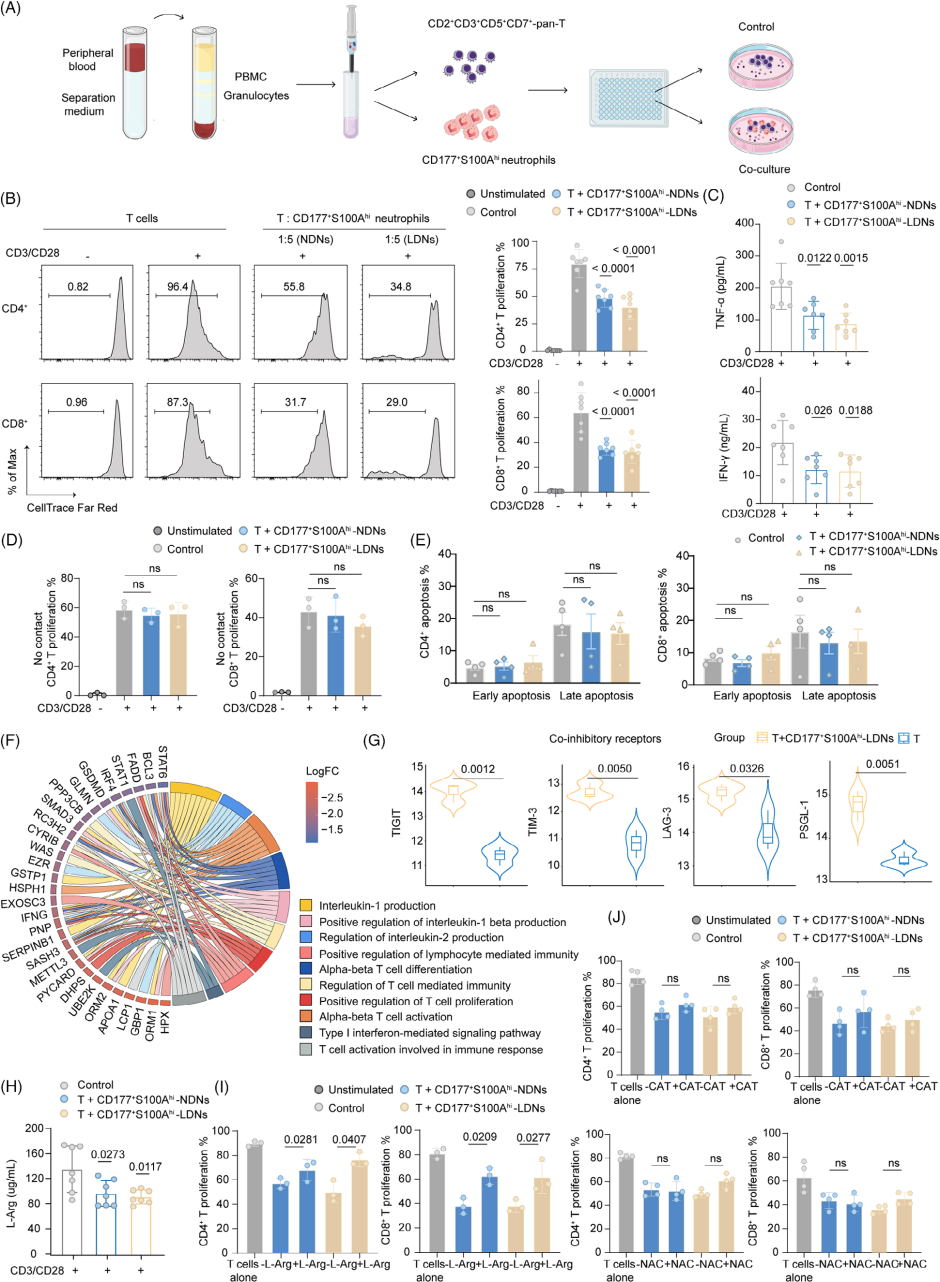
Immunosuppressive capacity of circulating CD177^+^S100A^hi^ neutrophils. (A) Schematic representation of T cells and neutrophils co‐culture system. CD3/CD28‐primed pan‐T cells from healthy donors were co‐cultured in the absence or presence of CD177^+^S100A^hi^ neutrophils from G‐PB of early pregnancy. (B and C) Representative images of proliferative alterations (B, left), proportion of proliferative CD4^+^ T cells (B, right top) and CD8^+^ T cells (B, right bottom), secretion of TNF‐α (C, top) and IFN‐γ (C, bottom) in co‐cultures of pan‐T cells with or without CD177^+^S100A^hi^‐LDNs and CD177^+^S100A^hi^‐NDNs (ratio of 1:5) at Day 3.5 (*n* = 7). Data are represented as mean ± SD. The symbol ‘–’ indicates T cells without CD3/CD28 stimulation, whereas ‘+’ designates T cells primed with CD3/CD28. (D) Boxplot showing proportion of proliferative CD4^+^ T cells (left) and CD8^+^ T cells (right) in co‐cultures of pan‐T cells with CD177^+^S100A^hi^‐LDNs and CD177^+^S100A^hi^‐NDNs (ratio of 1:5) under a Transwell device at Day 3.5 (*n* = 3). Data are represented as mean ± SD. (E) Quantification of apoptosis of CD4^+^ T cells and CD8^+^ T cells at a co‐culture ratio of 1:5 (*n* = 4). Data are represented as mean ± SEM. (F) Enriched GO terms for the downregulated proteins in T cells from co‐cultures of pan‐T cells with CD177^+^S100A^hi^‐LDNs. The thickness of the string is proportional to LogFC. (G) Violin plot of average expression of co‐inhibitory receptor molecules TIGIT, TIM‐3, LAG‐3 and PSGL‐1 in T cells exposed to CD177^+^S100A^hi^‐LDNs compared with cultured alone. The *y*‐axis represents the value of the log2 protein abundance. (H) L‐Arg concentration in supernatant from co‐cultures of pan‐T cells with or without CD177^+^S100A^hi^‐LDNs and CD177^+^S100A^hi^‐NDNs (ratio of 1:5) at Day 3.5 (*n* = 7). Data are represented as mean ± SD. (I and J) The proportion of proliferative CD4^+^ T cells (left) and CD8^+^ T cells (right) in co‐cultures of pan‐T cells with or without CD177^+^S100A^hi^‐LDNs and CD177^+^S100A^hi^‐NDNs (ratio of 1:5), with or without: 200 µg/mL L‐Arg (I); 200 U/mL CAT (J, top); 2 mM NAC (J, bottom). Data are represented as mean ± SD. *p*‐values are calculated using the one‐way ANOVA with Tukey's post hoc test and Student's *t*‐test. G‐PB, G‐CSF‐mobilised peripheral blood; LDNs, low density neutrophils; NDNs, normal density neutrophils; TNF‐α, tumour necrosis factor‐α; IFN‐γ, interferon‐γ; ARG1, arginase 1; ROS, reactive oxygen species; L‐Arg, L‐arginine; CAT, catalase.

Next, we further validate the CD177^+^S100A^hi^ neutrophil‐mediated immunosuppression in vitro. CD177⁺S100A^hi^ neutrophils, purified via magnetic‐activated cell sorting, were co‐cultured with allogeneic circulating T cells at a ratio of 5:1 (the most effective ratio), as illustrated in Figure 5A and Figure S7A–C. The result demonstrated that both CD177^+^S100A^hi^‐LDNs and CD177^+^S100A^hi^‐NDNs significantly impaired CD3/CD28‐primed proliferation, whether in CD4^+^ or CD8^+^ T lymphocytes (Figure 5B). T cells co‐cultured with CD177^+^S100A^hi^‐LDNs and CD177^+^S100A^hi^‐NDNs secreted less TNF‐α and IFN‐γ, compared with T cells cultured alone (Figure 5C). Crucially, this suppression was not detected when CD177^+^S100A^hi^ neutrophils and T cells were segregated via Transwells, suggesting that this inhibitory capacity was contact‐dependent rather than mediated by secreted signalling (Figure 5D). No significant apoptosis was observed in T cells cultured alone or exposed to neutrophils, demonstrating again that the impairment of T cells in G‐PB was not caused by apoptosis (Figure 5E; Figure S7D).

Both CD177⁺S100A^hi^‐LDNs and CD177⁺S100A^hi^‐NDNs suppressed T‐cell function. While their inhibitory capacities were statistically comparable, LDNs exhibited enhanced inhibition of proliferation and proinflammatory cytokine secretion. To dissect neutrophil‐mediated immunoregulation, we conducted comparative proteomic profiling between T cells cultured alone and exposed to CD177^+^S100A^hi^‐LDNs. Mass spectrometry analysis revealed that CD177^+^S100A^hi^‐LDNs substantially altered T‐cell proteomes (Figure S7E), with 915 proteins downregulated in co‐cultured T cells compared to those in T cells alone. Aligned with the previous observations, these downregulated proteins exhibited significant enrichment in pathways regulating T‐cell proliferation, differentiation, interleukin‐1 production and the Type I interferon‐mediated signalling pathway (Figure 5F; Figure S7F–H). Importantly, T cells exposed to CD177^+^S100A^hi^‐LDNs displayed coordinated upregulation of co‐inhibition molecules (TIGIT, TIM‐3, LAG‐3 and PSGL‐1),^43^, ^44^ alongside suppressed IFN‐γ expression (Figure 5G; Figure S7H). Notably, TNF‐α‐induced proteins were paradoxically upregulated in co‐cultured T cells (Figure S7I), possibly due to post‐transcriptional regulation. Although TNF‐α transcription is suppressed in G‐PB (Figure S5I), pre‐existing TNF‐α stores or residual signalling may sustain NF‐κB activation and induce negative regulators (SOCS1, SOCS3^45^) that inhibit secretion while preserving downstream signalling activation.^46^, ^47^ Furthermore, TNF‐α binding to TNFR1 could promote surface TNF‐α internalisation or preferential release of membrane‐bound forms—undetected in conventional soluble cytokine assays—potentially explaining the observed disparity.

We then investigated the mechanism of T‐cell suppression by CD177^+^S100A^hi^ neutrophils. A lower level of L‐Arg was detected in the supernatant of co‐cultures, and exogenous addition of L‐Arg could remarkably block the immunosuppressive effect induced by CD177^+^S100A^hi^ neutrophils, identifying ARG1 as a factor responsible for T‐cell suppression (Figure 5H,I). Moreover, when NAC (an ROS scavenger) or CAT was supplemented into co‐culture mediums, the number of T cells after their co‐culture with CD177^+^S100A^hi^ neutrophils was only slightly improved (Figure 5J). We concluded that CD177^+^S100A^hi^ neutrophils may form an ‘arginine‐deficient’ state in the local microenvironment through ARG1‐mediated L‐Arg depletion, thereby inducing T‐cell metabolic reprogramming and functional inhibition.

### 3.6 G‐CSF restored diminished CD177⁺S100A^hi^ neutrophils in the PB of patients with URPL, driving peripheral immunosuppression

**FIGURE 6:**
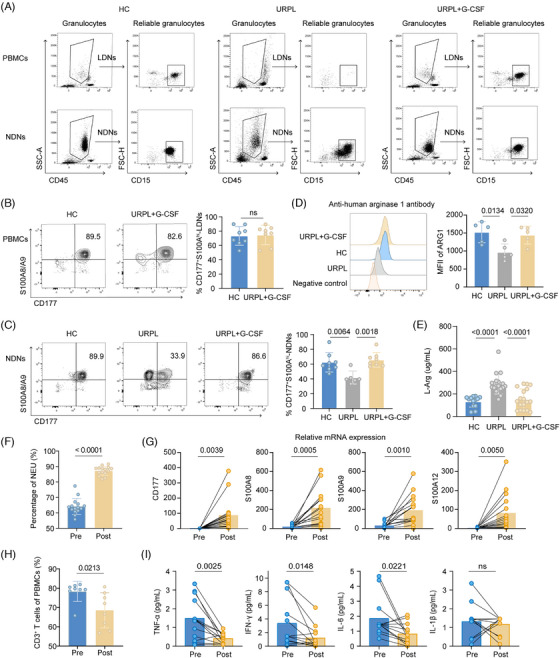
Diminished CD177^+^S100A^hi^ neutrophils in PB of URPL. (A) Representative flow cytometry plots showing distribution of neutrophils in PBMCs and NDNs layers of HCs, patients with URPL and URPL receiving G‐CSF treatment. NDNs and LDNs are gated based on CD45 and side‐scatter properties in PBMCs and NDNs fractions, respectively. Reliable neutrophils are identified as CD15^+^ cells among NDNs/LDNs. (B) Representative flow cytometry plots and quantification showing percentage of CD177^+^S100A^hi^‐LDNs in HCs and URPL patients receiving G‐CSF treatment (*n* = 8/each group). Data are represented as mean ± SD. (C) Representative flow cytometry plots and quantification showing percentage of CD177^+^S100A^hi^‐NDNs in HCs (*n* = 8), patients with URPL (*n* = 6) and URPL receiving G‐CSF treatment (*n* = 8). Data are represented as mean ± SD. (D) Flow cytometry of ARG1 expression in HC, patients with URPL and URPL receiving G‐CSF treatment (*n* = 5/each group). Data are represented as mean ± SD. (E) Serum L‐Arg concentrations in HCs (*n* = 13), patients with URPL (*n* = 18) and URPL receiving G‐CSF treatment (*n* = 18). Data are represented as mean ± SD. (F) The percentage of circulating neutrophils detected in PB before and after G‐CSF administration (*n* = 16/each group). (G) Relative mRNA expression of CD177, S100A8, S100A9 and S100A12 between G‐CSF‐mobilised and non‐mobilised neutrophils (*n* = 16/each group). (H) The percentage of circulating CD3^+^ T cells detected in PBMCs before and after G‐CSF administration (*n* = 8/each group). (I) Expression of inflammation‐related factors TNF‐α, IFN‐γ, IL‐6 and IL‐1β in pregnant women with URPL history before and after G‐CSF administration (*n* = 13/each group). *p*‐values are calculated using the one‐way ANOVA with Tukey's post hoc test and Student's *t*‐test. PBMCs, peripheral blood mononuclear cells; HCs, healthy pregnant controls; URPL, unexplained recurrent pregnancy loss; LDNs, low density neutrophils; NDNs, normal density neutrophils; ARG1, arginase 1; MFI, mean fluorescence intensity; L‐Arg, L‐arginine; TNF‐α, tumour necrosis factor‐α; IFN‐γ, interferon‐γ; IL‐6, interleukin‐6.

Considering the unique phenotype of CD177⁺S100A^hi^ neutrophils, we further explored their deficiency's association with maternal–fetal immune dysregulation, particularly in URPL. Flow cytometry of first‐trimester PB revealed a distinct distribution of CD177⁺S100A^hi^ neutrophils. Compared with HCs, CD177⁺S100A^hi^ neutrophils populated both PBMCs and NDNs layers, whereas URPL patients exhibited near‐complete depletion of this subpopulation in PBMC fractions and a significant reduction in NDN fractions, indicating compromised CD177⁺S100A^hi^ neutrophils in URPL in early gestation. Notably, administration of G‐CSF could restore the distribution of CD177⁺S100A^hi^ neutrophils in PBMC layers and normalise their proportion in NDN fractions to HC levels (Figure 6A–C; Figure S8A). In synchronisation with the expansion of CD177⁺S100A^hi^ neutrophils, we observed upregulated ARG1 expression and reduced serum L‐Arg concentrations in URPL patients after G‐CSF treatment, establishing G‐CSF‐mobilised CD177⁺S100A^hi^ neutrophils enhanced immune tolerance by modulating arginine metabolism during gestation (Figure 6D,E).

Moreover, neutrophils were purified via CD15 magnetic bead from PB before and after G‐CSF administration, confirming CD177⁺S100A^hi^ subpopulation responsiveness in a larger cohort. An increased percentage of circulating neutrophils was observed in G‐PB (Figure 6F), and there was a notable and unanimous upregulation of CD177 and S100A family genes (S100A8/A9/A12) in purified neutrophils following G‐CSF treatment (Figure 6G). In contrast, the proportion of T cells in the PBMCs decreased significantly (Figure 6H; Figure S8B). Proinflammatory factors such as TNF‐α, IFN‐γ and IL‐6 were markedly reduced in G‐PB. IL‐1β also revealed a downward tendency, though statistical thresholds were unmet (Figure 6I). These results were highly consistent with in vitro experimental findings, and demonstrated that G‐CSF induced early gestational immunosuppression through neutrophil‐T‐cell‐cytokine network remodelling.

### 3.7 G‐CSF restored reduced decidual CD177^+^S100A^hi^ neutrophils, rescuing pregnancy outcomes in AP mice

**FIGURE 7:**
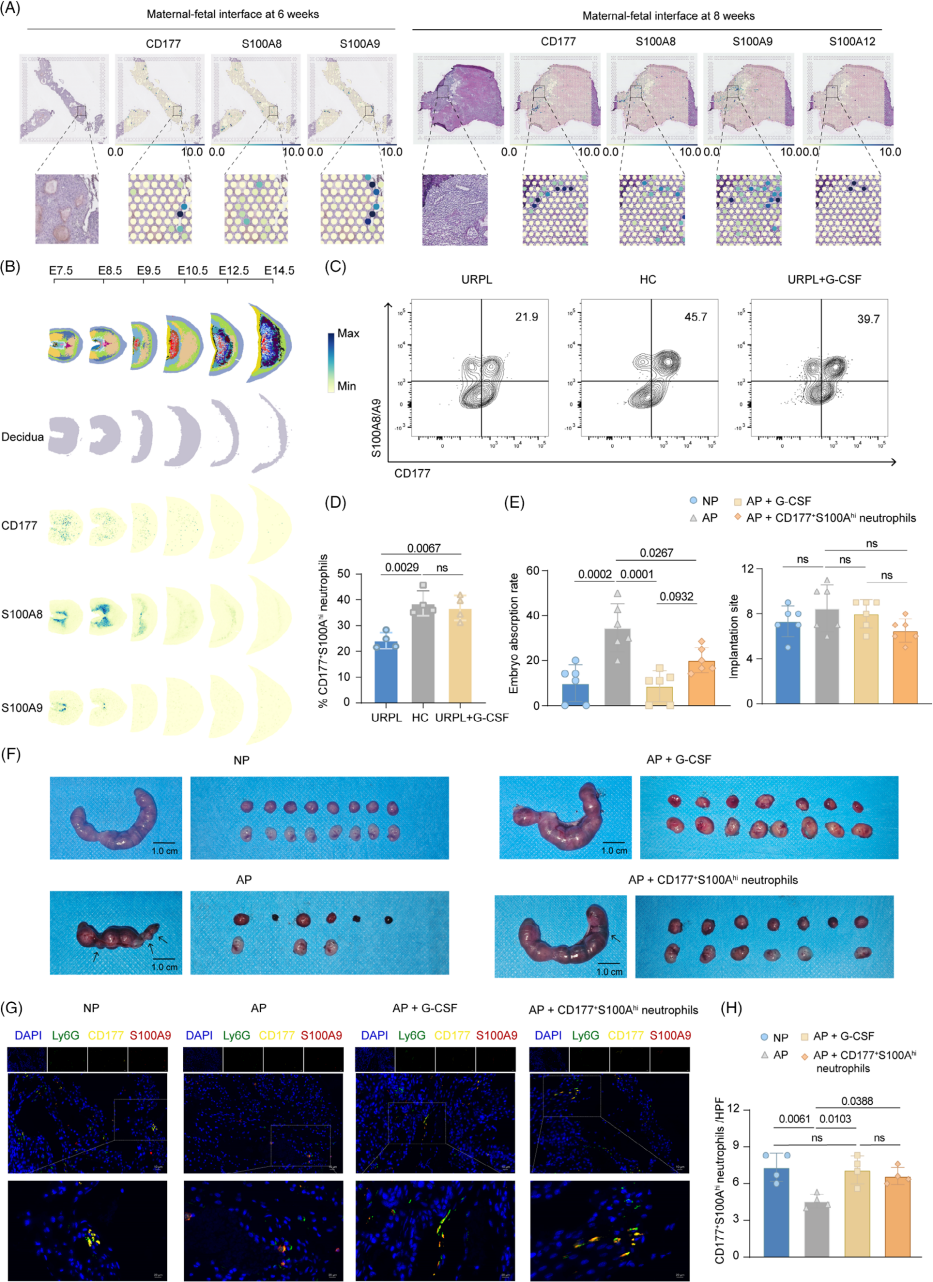
The protective role of CD177^+^S100A^hi^ neutrophils during pregnancy. (A) Spatial visualisation of CD177 and S100A family gene expression at the human maternal–fetal interface at 6 weeks (left) and 8 weeks (right). (B) Spatial visualisation of CD177 and S100A family gene expression at the mouse maternal–fetal interface at E7.5–E14.5. (C) Representative flow cytometry plots showing percentage of CD177^+^S100A^hi^ neutrophils in human decidual tissues of first trimester in HCs (*n* = 4), women with a history of URPL (*n* = 4) and URPL women receiving G‐CSF treatment (*n* = 4). (D) Quantification of CD177^+^S100A^hi^ neutrophils in human decidual tissues of first trimester in HCs (*n* = 4), women with a history of URPL (*n* = 4) and URPL women receiving G‐CSF treatment (*n* = 4). Data are represented as mean ± SD. (E) Quantification of embryo resorption rate (left) and implantation site (right) and in NP, AP, AP model treated with G‐CSF or CD177^+^S100A^hi^ neutrophils (each group: *n* = 6). Data are represented as mean ± SD. (F) Representative images of uteri and embryo in NP, AP, AP model treated with G‐CSF or CD177^+^S100A^hi^ neutrophils (each group: *n* = 6). Black arrows indicate embryo resorption. Scale bar: 1.0 cm. (G and H) Representative immunofluorescence images (G) and quantification (H) of CD177^+^S100A^hi^ neutrophils (defined as co‐expression of Ly6G, CD177 and S100A9) in NP, AP, AP model treated with G‐CSF or CD177^+^S100A^hi^ neutrophils (each group: *n* = 4). Scale bar: 20 µm, 10 µm. Data are represented as mean ± SD. *p*‐values are calculated using the one‐way ANOVA with Tukey's post hoc test. HCs, healthy pregnant controls; URPL, unexplained recurrent pregnancy loss; AP, abortion‐prone; NP, normal pregnancy; HPF, high‐power field.

At the maternal–fetal interface, we first examined the distribution of CD177⁺S100A^hi^ neutrophils using spatial transcriptome data. The spatiotemporal mapping atlas revealed the co‐localisation pattern of CD177 and S100A family genes at the maternal–fetal interface of healthy pregnant women, and their expression intensity was obviously upregulated at 8 weeks of gestation compared with 6 weeks of gestation (Figure 7A). The mouse placenta also showed consistent results. This tissue‐resident CD177^+^S100A^hi^ neutrophil was significantly enriched in the decidual region of early pregnancy, and its abundance gradually decreased with the progress of pregnancy (Figure 7B; Figure S9A).

Additionally, we collected decidual samples in the first trimester from HCs, patients with URPL and patients with URPL receiving G‐CSF treatment. Flow cytometry analysis demonstrated a pronounced reduction in CD177^+^S100A^hi^ neutrophil abundance within the decidua of URPL patients compared to HCs. G‐CSF‐treated URPL samples exhibited a significant restoration of CD177^+^S100A^hi^ neutrophils to levels comparable to those in HCs, consistent with the previous observations of PB (Figure 7C,D; Figure S9B).

In light of the observed immunomodulatory property, we established murine models of AP and NP to verify the role of G‐CSF and CD177^+^S100A^hi^ neutrophils. The detailed mating and treatment strategies are shown in Figure S9C. Compared to counterparts in the NP group, the AP group exhibited a higher embryo resorption rate, indicating successful model construction. In the treatment groups, exogenous supplementation with G‐CSF or adoptive transfer of CD177^+^S100A^hi^ neutrophils decreased embryo absorption rate to varying degrees (Figure 7E,F). G‐CSF exhibited the most obvious effect, but there was no statistically significant difference relative to supplementation with CD177^+^S100A^hi^ neutrophils. Moreover, compared with the NP group and the treatment group, the placental tissues from the AP group exhibited attenuated G‐CSF expression intensity, diminished CD177⁺S100A^hi^ neutrophil abundance, and prominent haemorrhagic foci (Figure 7G,H; Figure S9D,E). Collectively, these data indicate that G‐CSF and its derived CD177^+^S100A^hi^ neutrophils play undoubted positive roles during pregnancy in both people and mice.

## 4 DISCUSSION

G‐CSF, well‐known for its ability to mobilise and release granulocytes, has been reported to play a dual role in regulating immunity.^48^, ^49^ scRNA‐seq allowed us to deeply dissect the heterogeneous neutrophil population under C‐CSF stimulation.^28^ In our study, we identified a distinct population of CD177^+^S100A^hi^ neutrophils demonstrating PMN‐MDSCs characteristics. This specific subpopulation demonstrated significantly reduced abundance in both PB and decidual tissues of patients with URPL compared to HCs. Following G‐CSF administration, CD177^+^S100A^hi^ neutrophils were specifically expanded and induced T‐cell hyporesponsiveness through coordinated activation of the ARG1/L‐Arg metabolic axis and synergistic upregulation of immune checkpoint molecules, thereby reinforcing immune tolerance.

Neutrophils, as the major target cells of G‐CSF, possess considerable plasticity and heterogeneity. While neutrophils were traditionally characterised as ‘bad guys’ during pregnancy, the identification of MDSCs, specifically PMN‐MDSCs, has fundamentally challenged this paradigm. MDSCs were originally distinguished based on their capacity to suppress the proliferation of co‐cultured immune cells, particularly the main target T cells.^33^, ^50^ In the field of pregnancy, systematic investigations have demonstrated a pregnancy‐associated expansion of PMN‐MDSCs in both PB and uterine microenvironment,^51^, ^52^, ^53^ with multiple candidate surface markers, including CD11b^+^CD15^+^HLA‐DR^low^CD66b^+^,^54^ CD15^+^CD14^+^,^27^ CD66b^+^CD10^+^,^55^ CD33^+^CD11b^+^LOX‐1^+^.^56^ Herein, immunosuppressive CD177^+^S100A^hi^ neutrophils were identified based on discrepancies in transcriptomic‐proteomic profiles. They displayed PMN‐MDSC properties (immature morphology, elevated ROS and ARG1 activity and T‐cell suppression) and expanded substantially under G‐CSF stimulation. Similarly, a single‐cell dissection of human allogeneic haematopoietic stem and progenitor cells also found a unique population of immunoregulatory S100A^high^ neutrophil progenitors. The S100A^high^ neutrophil progenitors identified in this article also distinguished the remaining neutrophils by CD177.^57^

Corresponding with previous studies, CD177^+^S100A^hi^ neutrophils show a decrease in T‐cell proliferation and proinflammatory cytokine production.^55^, ^58^ Unlike conventional mixed co‐culture systems, our Transwell assay established strict physical segregation, which clearly revealed that the above‐mentioned T‐cell proliferation inhibitory effect strictly depends on direct contact between cells. Currently, persistent controversy surrounds the classification of PMN‐MDSCs, particularly regarding the maturation status of PMN‐MDSCs. A large number of studies identified PMN‐MDSCs as low‐density leukocytes exhibiting immature neutrophil morphology, predominantly localised in the PBMC fraction.^52^, ^59^, ^60^ On this basis, we integrated discontinuous Percoll gradient centrifugation (1.075/1.090 g/mL interfaces) with CD177 immunomagnetic sorting to acquire CD177^+^S100A^hi^ neutrophil subpopulations across density strata. Functional analysis showed that both CD177^+^S100A^hi^‐LDNs and CD177^+^S100A^hi^‐NDNs possessed suppressive abilities, with LDNs demonstrating superior immunosuppressive potency.

Although CD177⁺S100A^hi^ neutrophils and classical PMN‐MDSCs shared effector function implicated in suppressing T‐cell activation and proliferation, their molecular and phenotypic profiles are not entirely congruent. Recent studies have reaffirmed that the term ‘MDSC’ has been broadly applied to describe transcriptionally heterogeneous immune clusters.^54^ For example, lung carcinoma‐infiltrating subsets expressing TIE2,^61^ multiple myeloma‐infiltrating subsets expressing ITGAM, CD13 and CD16,^62^ and pancreatic ductal adenocarcinoma‐infiltrating subsets expressing ITGAM, S100A9 and APOE^63^ have been labelled as MDSCs, despite lacking unique defining markers. Importantly, the immunophenotypic characterisation of MDSCs remains an evolving field. Veglia et al. identified molecular markers differentiating PMN‐MDSCs from classical neutrophils, including OLR1 (encoding LOX‐1), STAT3, ARG1/2, S100A8/A9, CD244, and so forth.^34^ Among these, LOX‐1—a protein nearly undetectable in PB neutrophils of healthy individuals—has been established as a defining biomarker for PMN‐MDSCs in cancer patients.^64^ Intriguingly, our scRNA‐seq analyses revealed that OLR1, ARG1 and S100A8/A9 were also highly expressed in CD177⁺S100A^hi^ neutrophils, indicating partial molecular alignment between these cells and tumour‐associated PMN‐MDSCs. Morphologically, CD177⁺S100A^hi^‐LDNs exhibit a rounded nuclear architecture resembling that of N2‐polarised tumour‐associated neutrophils (which share hallmark features of PMN‐MDSCs),^29^, ^65^ in stark contrast to the multilobulated nuclei characteristic of terminally differentiated neutrophils, suggesting that CD177⁺S100A^hi^ neutrophils may represent a unique transcriptional population within the broader PMN‐MDSCs framework.

The mechanisms underlying the outcome of PMN‐MDSCs‐T‐cell crosstalk have been extensively investigated. In addition to the cell activation status, local soluble mediators are increasingly recognised as pivotal influencing factors.^66^, ^67^ Multiple immunosuppressive mediators, including ARG1, ROS, PD‐L1, indoleamine 2,3‐dioxygenase, TGF‐β and IL10, have been reported to participate in neutrophil‐T‐cell crosstalk.^34^, ^59^, ^68^, ^69^ Among these, neutrophil‐derived ROS and ARG1 are the most classical and are also considered to be related to the inhibitory activity of PMN‐MDSCs.^68^ ^,^ ^70^, ^71^ Our data showed that isolated CD177^+^S100A^hi^ neutrophils exhibited a higher expression of ARG1 and ROS. The enhancement of ARG1 activity during pregnancy supports immune tolerance at the maternal–fetal interface by efficiently depleting arginine in the surroundings (hydrolysing arginine into ornithine and urea). This process suppresses T‐cell expansion without augmenting T‐cell apoptosis or death.^72^ Arginine depletion also downregulates the CD3ζ chain and impairs TCR signalling as well as synapse formation, as confirmed in our single‐cell transcriptome and assembled TCR repertoire.^72^, ^73^, ^74^ ROS has also been linked with T‐cell metabolic reprogramming. Prolonged ROS signalling causes T‐cell hyporesponsiveness via targeted impairment of TCR‐dependent PLC‐γ1 phosphorylation,^75^ and higher exposure may result in T‐cell apoptosis or necrosis.^76^, ^77^ In this study, T‐cell proliferation was rescued by exogenous supplementation with L‐Arg instead of NAC and CAT. Integrated with the absence of significant differences in T‐cell apoptosis rates in the co‐culture system, our findings demonstrated that CD177^+^S100A^hi^ neutrophil‐derived ARG1, rather than ROS, exerted a crucial role in mediating T‐cell dysfunction, which is different from a previous study that G‐CSF induced CD15^+^CD14^+^ cells impaired T‐cell expansion via ROS.^27^

Immune homeostasis dysregulation served as a pathogenic mechanism underlying adverse pregnancy outcomes, particularly prominent in URPL. Unlike the systemic immunosuppression characteristic observed in healthy pregnancy, URPL patients exhibited a peripheral proinflammatory state marked by compromised PMN‐MDSCs and T‐cell hyperactivation.^78^, ^79^, ^80^ Our analysis identified substantial depletion of immunosuppressive CD177^+^S100A^hi^ neutrophils in URPL PB (PBMC/NDN fractions). Notably, G‐CSF administration stimulated expansion of this subpopulation while simultaneously suppressing CD3⁺T‐cell proliferation and downregulating inflammatory cytokines, thereby ameliorating the pathological inflammatory state. We further focused on the immune microenvironment of the decidual tissue at the maternal–fetal interface. The abundance of CD177⁺S100A^hi^ neutrophils was markedly reduced in URPL patients, suggesting impaired maternal–fetal immune tolerance may correlate with the deficiency of CD177^+^S100A^hi^ neutrophils. However, crucially, decidual tissue from URPL patients was obtained post‐embryonic arrest, rendering the causation between the reduction of CD177^+^S100A^hi^ neutrophils and pregnancy loss indeterminate.

To address this limitation, we employed AP mouse models and administered therapeutic interventions at gestational day 4.5, a critical window corresponding to both embryo implantation and the initiation of maternal–fetal immune tolerance.^24^, ^81^ Functional validation in AP mice demonstrated that both G‐CSF administration and adoptive transfer of CD177⁺S100A^hi^ neutrophils reduced fetal resorption rates. These findings collectively indicated that G‐CSF‐mobilised CD177⁺S100A^hi^ neutrophils constitute a critical immunoregulatory mechanism sustaining fetal–maternal tolerance, with their deficiency potentially initiating a pathological cascade, ultimately leading to pregnancy failure.

Sampling and ethical considerations limited the exploration of immune cells, especially neutrophils. Although animal experiments suggest its functional importance, the dynamic changes in the human decidual microenvironment may also be regulated by the feedback of the embryonic developmental state. Some scholars proposed that menstrual blood variations better reflected uterine dynamics than PB, but there was a lack of consensus on this view, and controversy still existed regarding the optimal sampling period.^82^, ^83^ Validation employing conditional knockout models or longitudinal cohort studies remained critical to delineate spatiotemporal regulatory networks. Our study found that immunosuppressive CD177^+^S100A^hi^ neutrophils increased in G‐PB, which induced T‐cell hyporesponsiveness. But this work cannot exclude the role of CD177^+^S100A^hi^ neutrophils in interaction with other cells at the maternal–fetal interface, such as macrophages, which are mainly distributed in tissues rather than in PB. While the current cohort demonstrated sufficient statistical power to identify G‐CSF‐associated cellular changes, future investigations would employ expanded clinical cohorts and gene knockout to validate translational relevance. These multidimensional validation frameworks will systematically resolve biological mechanisms underlying treatment responses while addressing generalisability limitations.

## AUTHOR CONTRIBUTIONS

**Ping‐fen Li**: investigation, data curation, methodology, formal analysis, validation, writing—original draft. **Xue Zhang**: investigation, data curation. **Peng‐Sheng Zheng**: funding acquisition, project administration, resources, supervision, writing—review and editing.

## CONFLICT OF INTEREST STATEMENT

The authors declare they have no conflicts of interest.

## ETHICS STATEMENT

This research adheres to the highest ethical standards, aligning with the principles outlined in the Declaration of Helsinki. All human sampling was in accordance with the Declaration of Helsinki and approved by the Institutional Review Board of the Ethics Committee of the School of Medicine, Xi'an Jiaotong University (XJTU1AF2024LSYY‐430). All participants were fully informed about the study and provided signed consent forms. All mouse experiments were conducted in accordance with the ‘the Animal Research: Reporting of In Vivo Experiments’ (ARRIVE) and ‘Principles of Experimental Animal Care’ (NIH) guidelines at the Laboratory Animal Center, and were approved by the Animal Ethics Committee of Xi'an Jiaotong University (XJTU1AE2025‐713).

## Supporting information



Supporting Information

## Data Availability

The datasets utilised and/or analysed during this study can be obtained from the corresponding author upon reasonable request.

## References

[1] Mor G . Introduction to the immunology of pregnancy. Immunol Rev. 2022;308:5‐8. doi:10.1111/imr.13102 35635382 10.1111/imr.13102

[2] Xu L , Li Y , Sang Y , Li DJ , Du M . Crosstalk between trophoblasts and decidual immune cells: the cornerstone of maternal‐fetal immunotolerance. Front Immunol. 2021;12:642392. doi:10.3389/fimmu.2021.642392 33717198 10.3389/fimmu.2021.642392 PMC7947923

[3] Bender Atik R , Christiansen OB , Elson J , et al. ESHRE guideline: recurrent pregnancy loss. Human Reprod Open. 2018;2018:hoy004. 10.1093/hropen/hoy004 PMC6276652 31486805

[4] Definitions of infertility and recurrent pregnancy loss: a committee opinion. Fertil Steril. 2020;113:533‐535. doi:10.1016/j.fertnstert.2019.11.025 32115183 10.1016/j.fertnstert.2019.11.025

[5] Regan L , Rai R . Epidemiology and the medical causes of miscarriage. Best Pract Res Clin Obstet Gynaecol. 2000;14:839‐854. doi:10.1053/beog.2000.0123 10.1053/beog.2000.0123 11023804

[6] Cramer DW , Wise LA . The epidemiology of recurrent pregnancy loss. Semin Reprod Med. 2000;18:331‐339. doi:10.1055/s‐2000‐13722 11355791 10.1055/s-2000-13722

[7] Eftekhar M , Naghshineh E , Khani P . Role of granulocyte colony‐stimulating factor in human reproduction. J Res Med Sci. 2018;23:7. 29456564 10.4103/jrms.JRMS_628_17 PMC5813296

[8] Cavalcante MB , Costa Fda S , Barini R , Araujo Júnior E . Granulocyte colony‐stimulating factor and reproductive medicine: a review. Iran J Reprod Med. 2015;13:195‐202. 26131007 PMC4475767

[9] Ding J , Wang J , Cai X , et al. Granulocyte colony‐stimulating factor in reproductive‐related disease: function, regulation and therapeutic effect. Biomed Pharmacother. 2022;150:112903. doi:10.1016/j.biopha.2022.112903 35430390 10.1016/j.biopha.2022.112903

[10] Gao P , Zha Y , Wei L , et al. A vehicle for communication between trophoblasts and macrophages which may cause problems in recurrent spontaneous abortion. Placenta. 2022;121:164‐172. doi:10.1016/j.placenta.2022.03.125 35364512 10.1016/j.placenta.2022.03.125

[11] Scarpellini F , Klinger FG , Rossi G , Sbracia M . Immunohistochemical study on the expression of G‐CSF, G‐CSFR, VEGF, VEGFR‐1, Foxp3 in first trimester trophoblast of recurrent pregnancy loss in pregnancies treated with G‐CSF and controls. Int J Mol Sci. 2019;21:285. doi:10.3390/ijms21010285 31906232 10.3390/ijms21010285 PMC6981573

[12] Scarpellini F , Sbracia M . Use of granulocyte colony‐stimulating factor for the treatment of unexplained recurrent miscarriage: a randomised controlled trial. Hum Reprod. 2009;24:2703‐2708. doi:10.1093/humrep/dep240 19617208 10.1093/humrep/dep240

[13] Santjohanser C , Knieper C , Franz C , et al. Granulocyte‐colony stimulating factor as treatment option in patients with recurrent miscarriage. Arch Immunol Ther Exp (Warsz). 2013;61:159‐164. doi:10.1007/s00005‐012‐0212‐z 23344173 10.1007/s00005-012-0212-z

[14] Mu F , Huang J , Zeng X , Liu L , Wang F . Efficacy and safety of recombinant human granulocyte colony‐stimulating factor in patients with unexplained recurrent spontaneous abortion: a systematic review and meta‐analysis. J Reprod Immunol. 2023;156:103830. doi:10.1016/j.jri.2023.103830 36821985 10.1016/j.jri.2023.103830

[15] Shao Q , Liu X , Huang Y , Chen X , Wang H . Human decidual stromal cells in early pregnancy induce functional re‐programming of monocyte‐derived dendritic cells via crosstalk between G‐CSF and IL‐1β. Front Immunol. 2020;11:574270. doi:10.3389/fimmu.2020.574270 33193360 10.3389/fimmu.2020.574270 PMC7652738

[16] Li W , Zhang X , Chen Y , et al. G‐CSF is a key modulator of MDSC and could be a potential therapeutic target in colitis‐associated colorectal cancers. Protein Cell. 2016;7:130‐140. doi:10.1007/s13238‐015‐0237‐2 26797765 10.1007/s13238-015-0237-2 PMC4742385

[17] Abrams SI , Waight JD . Identification of a G‐CSF‐granulocytic MDSC axis that promotes tumour progression. Oncoimmunology. 2012;1:550‐551. doi:10.4161/onci.19334 22754783 10.4161/onci.19334 PMC3382879

[18] Schmid KT , Höllbacher B , Cruceanu C , et al. scPower accelerates and optimizes the design of multi‐sample single cell transcriptomic studies. Nat Commun. 2021;12:6625. doi:10.1038/s41467‐021‐26779‐7 34785648 10.1038/s41467-021-26779-7 PMC8595682

[19] Hu XE , Yang P , Chen S , et al. Clinical and biological heterogeneities in triple‐negative breast cancer reveals a non‐negligible role of HER2‐low. Breast Cancer Res. 2023;25:34. doi:10.1186/s13058‐023‐01639‐y 36998014 10.1186/s13058-023-01639-y PMC10061837

[20] Li QS , Zheng PS . ESRRB inhibits the TGFβ signaling pathway to drive cell proliferation in cervical cancer. Cancer Res. 2023;83:3095‐3114. doi:10.1158/0008‐5472.CAN‐23‐0067 37350664 10.1158/0008-5472.CAN-23-0067 PMC10502452

[21] Lei D , Yang WT , Zheng PS . HOXB4 inhibits the proliferation and tumourigenesis of cervical cancer cells by downregulating the activity of Wnt/β‐catenin signaling pathway. Cell Death Dis. 2021;12:105. doi:10.1038/s41419‐021‐03411‐6 33479226 10.1038/s41419-021-03411-6 PMC7820415

[22] Wang G , Wu S , Xiong Z , Qu H , Fang X , Bao Y . CROST: a comprehensive repository of spatial transcriptomics. Nucleic Acids Res. 2024;52:D882‐D890. doi:10.1093/nar/gkad782 37791883 10.1093/nar/gkad782 PMC10773281

[23] Wu Y , Su K , Zhang Y , et al. A spatiotemporal transcriptomic atlas of mouse placentation. Cell Discov. 2024;10:110. doi:10.1038/s41421‐024‐00740‐6 39438452 10.1038/s41421-024-00740-6 PMC11496649

[24] Yang M , Ong J , Meng F , et al. Spatiotemporal insight into early pregnancy governed by immune‐featured stromal cells. Cell. 2023;186:4271‐4288.e24. doi:10.1016/j.cell.2023.08.020 37699390 10.1016/j.cell.2023.08.020

[25] Wang J , Yang J , Yan Y , et al. Effect of adoptive transfer of CD4(+)CD25(+)Foxp3(+) Treg induced by trichostatin A on the prevention of spontaneous abortion. J Reprod Immunol. 2019;131:30‐35. doi:10.1016/j.jri.2018.12.002 30634133 10.1016/j.jri.2018.12.002

[26] Jia W , Ma L , Yu X , et al. Human CD56(+)CD39(+) dNK cells support fetal survival through controlling trophoblastic cell fate: immune mechanisms of recurrent early pregnancy loss. Natl Sci Rev. 2024;11:nwae142. doi:10.1093/nsr/nwae142 38966071 10.1093/nsr/nwae142 PMC11223582

[27] Maneta E , Fultang L , Taylor J , et al. G‐CSF induces CD15(+) CD14(+) cells from granulocytes early in the physiological environment of pregnancy and the cancer immunosuppressive microenvironment. Clin Transl Immunol. 2022;11:e1395. doi:10.1002/cti2.1395 10.1002/cti2.1395 PMC9114661 35602884

[28] Xie X , Shi Q , Wu P , et al. Single‐cell transcriptome profiling reveals neutrophil heterogeneity in homeostasis and infection. Nat Immunol. 2020;21:1119‐1133. doi:10.1038/s41590‐020‐0736‐z 32719519 10.1038/s41590-020-0736-z PMC7442692

[29] Silvestre‐Roig C , Fridlender ZG , Glogauer M , Scapini P . Neutrophil diversity in health and disease. Trends Immunol. 2019;40:565‐583. doi:10.1016/j.it.2019.04.012 31160207 10.1016/j.it.2019.04.012 PMC7185435

[30] Schneider WM , Chevillotte MD , Rice CM . Interferon‐stimulated genes: a complex web of host defenses. Annu Rev Immunol. 2014;32:513‐545. doi:10.1146/annurev‐immunol‐032713‐120231 24555472 10.1146/annurev-immunol-032713-120231 PMC4313732

[31] Ding J , Maxwell A , Adzibolosu N , et al. Mechanisms of immune regulation by the placenta: role of type I interferon and interferon‐stimulated genes signaling during pregnancy. Immunol Rev. 2022;308:9‐24. doi:10.1111/imr.13077 35306673 10.1111/imr.13077 PMC9189063

[32] Evrard M , Kwok IWH , Chong SZ , et al. Developmental analysis of bone marrow neutrophils reveals populations specialized in expansion, trafficking, and effector functions. Immunity. 2018;48:364‐379.e8. doi:10.1016/j.immuni.2018.02.002 29466759 10.1016/j.immuni.2018.02.002

[33] Ikeda N , Kubota H , Suzuki R , et al. The early neutrophil‐committed progenitors aberrantly differentiate into immunoregulatory monocytes during emergency myelopoiesis. Cell Rep. 2023;42:112165. doi:10.1016/j.celrep.2023.112165 36862552 10.1016/j.celrep.2023.112165

[34] Veglia F , Sanseviero E , Gabrilovich DI . Myeloid‐derived suppressor cells in the era of increasing myeloid cell diversity. Nat Rev Immunol. 2021;21:485‐498. doi:10.1038/s41577‐020‐00490‐y 33526920 10.1038/s41577-020-00490-y PMC7849958

[35] von Wulffen M , Luehrmann V , Robeck S , et al. S100A8/A9‐alarmin promotes local myeloid‐derived suppressor cell activation restricting severe autoimmune arthritis. Cell Rep. 2023;42:113006. doi:10.1016/j.celrep.2023.113006 37610870 10.1016/j.celrep.2023.113006

[36] Li K , Shi H , Zhang B , et al. Myeloid‐derived suppressor cells as immunosuppressive regulators and therapeutic targets in cancer. Signal Transduct Target Ther. 2021;6:362. doi:10.1038/s41392‐021‐00670‐9 34620838 10.1038/s41392-021-00670-9 PMC8497485

[37] Zhou J , Nefedova Y , Lei A . Neutrophils and PMN‐MDSC: their biological role and interaction with stromal cells. Semin Immunol. 2018;35:19‐28. doi:10.1016/j.smim.2017.12.004 29254756 10.1016/j.smim.2017.12.004 PMC5866202

[38] Nguyen CT , Furuya H , Das D , et al. Peripheral γδ T cells regulate neutrophil expansion and recruitment in experimental psoriatic arthritis. Arthritis Rheumatol (Hoboken, NJ). 2022;74:1524‐1534. doi:10.1002/art.42124 10.1002/art.42124 PMC9427669 35320625

[39] Pelletier M , Maggi L , Micheletti A , et al. Evidence for a cross‐talk between human neutrophils and Th17 cells. Blood. 2010;115:335‐343. doi:10.1182/blood‐2009‐04‐216085 19890092 10.1182/blood-2009-04-216085

[40] Hirschhorn D , Budhu S , Kraehenbuehl L , et al. T‐cell immunotherapies engage neutrophils to eliminate tumour antigen escape variants. Cell. 2023;186:1432‐1447.e17. doi:10.1016/j.cell.2023.03.007 37001503 10.1016/j.cell.2023.03.007 PMC10994488

[41] Aoki H , Shichino S , Matsushima K , Ueha S . Revealing clonal responses of tumour‐reactive T‐cells through T cell receptor repertoire analysis. Front Immunol. 2022;13:807696. doi:10.3389/fimmu.2022.807696 35154125 10.3389/fimmu.2022.807696 PMC8829044

[42] Pai JA , Satpathy AT . High‐throughput and single‐cell T‐cell receptor sequencing technologies. Nat Methods. 2021;18:881‐892. doi:10.1038/s41592‐021‐01201‐8 34282327 10.1038/s41592-021-01201-8 PMC9345561

[43] Tinoco R , Otero DC , Takahashi AA , Bradley LM . PSGL‐1: a new player in the immune checkpoint landscape. Trends Immunol. 2017;38:323‐335. doi:10.1016/j.it.2017.02.002 28262471 10.1016/j.it.2017.02.002 PMC5411281

[44] Tinoco R , Carrette F , Barraza ML , et al. PSGL‐1 is an immune checkpoint regulator that promotes T cell exhaustion. Immunity. 2016;44:1190‐1203. doi:10.1016/j.immuni.2016.04.015 27192578 10.1016/j.immuni.2016.04.015 PMC4908967

[45] Collins AS , Ahmed S , Napoletano S , et al. Hepatitis C virus (HCV)‐induced suppressor of cytokine signaling (SOCS) 3 regulates proinflammatory TNF‐α responses. J Leukocyte Biol. 2014;96:255‐263. doi:10.1189/jlb.2A1211‐608RRRR 24659790 10.1189/jlb.2A1211-608RRRR

[46] Jia L , Shi Y , Wen Y , Li W , Feng J , Chen C . The roles of TNFAIP2 in cancers and infectious diseases. J Cell Mol Med. 2018;22:5188‐5195. doi:10.1111/jcmm.13822 30145807 10.1111/jcmm.13822 PMC6201362

[47] Huyghe J , Priem D , Bertrand MJM . Cell death checkpoints in the TNF pathway. Trends Immunol. 2023;44:628‐643. doi:10.1016/j.it.2023.05.007 37357102 10.1016/j.it.2023.05.007

[48] Mehta HM , Corey SJ . G‐CSF, the guardian of granulopoiesis. Semin Immunol. 2021;54:101515. doi:10.1016/j.smim.2021.101515 34772606 10.1016/j.smim.2021.101515

[49] Martin KR , Wong HL , Witko‐Sarsat V , Wicks IP . G‐CSF—a double edge sword in neutrophil mediated immunity. Semin Immunol. 2021;54:101516. doi:10.1016/j.smim.2021.101516 34728120 10.1016/j.smim.2021.101516

[50] Bennett JA , Rao VS , Mitchell MS . Systemic bacillus Calmette‐Guérin (BCG) activates natural suppressor cells. Proc Natl Acad Sci U.S.A. 1978;75:5142‐5144. doi:10.1073/pnas.75.10.5142 283421 10.1073/pnas.75.10.5142 PMC336280

[51] Zheng ZM , Yang HL , Lai ZZ , et al. Myeloid‐derived suppressor cells in obstetrical and gynecological diseases. Am J Reprod Immunol. 2020;84:e13266. doi:10.1111/aji.13266 32418253 10.1111/aji.13266

[52] Köstlin N , Kugel H , Spring B , et al. Granulocytic myeloid derived suppressor cells expand in human pregnancy and modulate T‐cell responses. Eur J Immunol. 2014;44:2582‐2591. 24894988 10.1002/eji.201344200

[53] Zhao AM , Xu HJ , Kang XM , Zhao AM , Lu LM . New insights into myeloid‐derived suppressor cells and their roles in feto‐maternal immune cross‐talk. J Reprod Immunol. 2016;113:35‐41. doi:10.1016/j.jri.2015.11.001 26599285 10.1016/j.jri.2015.11.001

[54] Hegde S , Leader AM , Merad M . MDSC: markers, development, states, and unaddressed complexity. Immunity. 2021;54:875‐884. doi:10.1016/j.immuni.2021.04.004 33979585 10.1016/j.immuni.2021.04.004 PMC8709560

[55] Marini O , Costa S , Bevilacqua D , et al. Mature CD10(+) and immature CD10(−) neutrophils present in G‐CSF‐treated donors display opposite effects on T cells. Blood. 2017;129:1343‐1356. doi:10.1182/blood‐2016‐04‐713206 28053192 10.1182/blood-2016-04-713206

[56] Li C , Chen C , Kang X , et al. Decidua‐derived granulocyte macrophage colony‐stimulating factor induces polymorphonuclear myeloid‐derived suppressor cells from circulating CD15^+^ neutrophils. Hum Reprod. 2020;35:2677‐2691. doi:10.1093/humrep/deaa217 33067638 10.1093/humrep/deaa217

[57] Huo Y , Wu L , Pang A , et al. Single‐cell dissection of human hematopoietic reconstitution after allogeneic hematopoietic stem cell transplantation. Sci Immunol. 2023;8:eabn6429. doi:10.1126/sciimmunol.abn6429 36930730 10.1126/sciimmunol.abn6429

[58] Zhao X , Peng T , Cao X , et al. In vivo G‐CSF treatment activates the GR‐SOCS1 axis to suppress IFN‐γ secretion by natural killer cells. Cell Rep. 2022;40:111342. doi:10.1016/j.celrep.2022.111342 36103837 10.1016/j.celrep.2022.111342

[59] Veglia F , Perego M , Gabrilovich D . Myeloid‐derived suppressor cells coming of age. Nat Immunol. 2018;19:108‐119. doi:10.1038/s41590‐017‐0022‐x 29348500 10.1038/s41590-017-0022-x PMC5854158

[60] Shi M , Chen Z , Chen M , et al. Continuous activation of polymorphonuclear myeloid‐derived suppressor cells during pregnancy is critical for fetal development. Cell Mol Immunol. 2021;18:1692‐1707. doi:10.1038/s41423‐021‐00704‐w 34099889 10.1038/s41423-021-00704-w PMC8245399

[61] Lauret Marie , Joseph E , Laheurte C , et al. Immunoregulation and clinical implications of ANGPT2/TIE2(+) M‐MDSC signature in non‐small cell lung cancer. Cancer Immunol Res. 2020;8:268‐279. doi:10.1158/2326‐6066.CIR‐19‐0326 31871121 10.1158/2326-6066.CIR-19-0326

[62] Perez C , Botta C , Zabaleta A , et al. Immunogenomic identification and characterization of granulocytic myeloid‐derived suppressor cells in multiple myeloma. Blood. 2020;136:199‐209. doi:10.1182/blood.2019004537 32325491 10.1182/blood.2019004537

[63] Bernard V , Semaan A , Huang J , et al. Single‐cell transcriptomics of pancreatic cancer precursors demonstrates epithelial and microenvironmental heterogeneity as an early event in neoplastic progression. 2019;25:2194‐2205. doi:10.1158/1078‐0432.CCR‐18‐1955 10.1158/1078-0432.CCR-18-1955 PMC6445737 30385653

[64] Condamine T , Dominguez GA , Youn JI , et al. Lectin‐type oxidized LDL receptor‐1 distinguishes population of human polymorphonuclear myeloid‐derived suppressor cells in cancer patients. Sci Immunol. 2016;1:aaf8943. doi:10.1126/sciimmunol.aaf8943 28417112 10.1126/sciimmunol.aaf8943 PMC5391495

[65] Huang C , Fan X , Shen Y , Shen M , Yang L . Neutrophil subsets in noncancer liver diseases: cellular crosstalk and therapeutic targets. Eur J Immunol. 2023;53:e2250324. doi:10.1002/eji.202250324 37495829 10.1002/eji.202250324

[66] Minns D , Smith KJ , Hardisty G , Rossi AG , Gwyer Findlay E . The outcome of neutrophil‐T cell contact differs depending on activation status of both cell types. Front Immunol. 2021;12:633486. doi:10.3389/fimmu.2021.633486 33859639 10.3389/fimmu.2021.633486 PMC8042376

[67] Michaeli J , Shaul ME , Mishalian I , et al. Tumour‐associated neutrophils induce apoptosis of non‐activated CD8 T‐cells in a TNFα and NO‐dependent mechanism, promoting a tumour‐supportive environment. Oncoimmunology. 2017;6:e1356965. doi:10.1080/2162402X.2017.1356965 29147615 10.1080/2162402X.2017.1356965 PMC5674962

[68] Gabrilovich DI . Myeloid‐derived suppressor cells. Cancer Immunol Res. 2017;5:3‐8. doi:10.1158/2326‐6066.CIR‐16‐0297 28052991 10.1158/2326-6066.CIR-16-0297 PMC5426480

[69] Bert S , Nadkarni S , Perretti M . Neutrophil‐T‐cell crosstalk and the control of the host inflammatory response. Immunol Rev. 2023;314:36‐49. doi:10.1111/imr.13162 36326214 10.1111/imr.13162 PMC10952212

[70] Geiger R , Rieckmann JC , Wolf T , et al. L‐Arginine modulates T cell metabolism and enhances survival and anti‐tumour activity. Cell. 2016;167:829‐842.e13. doi:10.1016/j.cell.2016.09.031 27745970 10.1016/j.cell.2016.09.031 PMC5075284

[71] Morris G , Gevezova M , Sarafian V , Maes M . Redox regulation of the immune response. Cell Mol Immunol. 2022;19:1079‐1101. doi:10.1038/s41423‐022‐00902‐0 36056148 10.1038/s41423-022-00902-0 PMC9508259

[72] Munder M , Schneider H , Luckner C , et al. Suppression of T‐cell functions by human granulocyte arginase. Blood. 2006;108:1627‐1634. doi:10.1182/blood‐2006‐11‐010389 16709924 10.1182/blood-2006-11-010389

[73] Rodriguez PC , Zea AH , Culotta KS , Zabaleta J , Ochoa JB , Ochoa AC . Regulation of T‐cell receptor CD3zeta chain expression by L‐arginine. J Biol Chem. 2002;277:21123‐21129. doi:10.1074/jbc.M110675200 11950832 10.1074/jbc.M110675200

[74] Feldmeyer N , Wabnitz G , Leicht S , et al. Arginine deficiency leads to impaired cofilin dephosphorylation in activated human T lymphocytes. Int Immunol. 2012;24:303‐313. doi:10.1093/intimm/dxs004 22345165 10.1093/intimm/dxs004

[75] Cemerski S , Cantagrel A , Van Meerwijk JP , Romagnoli P . Reactive oxygen species differentially affect T‐cell receptor‐signaling pathways. J Biol Chem. 2002;277:19585‐19593. doi:10.1074/jbc.M111451200 11916964 10.1074/jbc.M111451200

[76] Sies H , Jones DP . Reactive oxygen species (ROS) as pleiotropic physiological signalling agents. Nat Rev Mol Cell Biol. 2020;21:363‐383. doi:10.1038/s41580‐020‐0230‐3 32231263 10.1038/s41580-020-0230-3

[77] Franchina DG , Dostert C , Brenner D . Reactive oxygen species: involvement in T cell signaling and metabolism. Trends Immunol. 2018;39:489‐502. doi:10.1016/j.it.2018.01.005 29452982 10.1016/j.it.2018.01.005

[78] Chen D , Wang W , Wu L , et al. Single‐cell atlas of peripheral blood mononuclear cells from pregnant women. Clin Transl Med. 2022;12:e821. doi:10.1002/ctm2.821 35522918 10.1002/ctm2.821 PMC9076016

[79] Li C , Zhang X , Kang X , et al. Upregulated TRAIL and reduced DcR2 mediate apoptosis of decidual PMN‐MDSC in unexplained recurrent pregnancy loss. Front Immunol. 2020;11:1345. doi:10.3389/fimmu.2020.01345 32695113 10.3389/fimmu.2020.01345 PMC7338483

[80] Qin D , Xu H , Chen Z , et al. The peripheral and decidual immune cell profiles in women with recurrent pregnancy loss. Front Immunol. 2022;13:994240. doi:10.3389/fimmu.2022.994240 36177021 10.3389/fimmu.2022.994240 PMC9513186

[81] Sojka DK , Yang L , Yokoyama WM . Uterine natural killer cells. Front Immunol. 2019;10:960. doi:10.3389/fimmu.2019.00960 31118936 10.3389/fimmu.2019.00960 PMC6504766

[82] Tong X , Gao M , Du X , et al. Analysis of uterine CD49a(+) NK cell subsets in menstrual blood reflects endometrial status and association with recurrent spontaneous abortion. Cell Mol Immunol. 2021;18:1838‐1840. doi:10.1038/s41423‐021‐00687‐8 34002045 10.1038/s41423-021-00687-8 PMC8245401

[83] van der Molen RG , Schutten JH , van Cranenbroek B , et al. Menstrual blood closely resembles the uterine immune micro‐environment and is clearly distinct from peripheral blood. Hum Reprod. 2014;29:303‐314. doi:10.1093/humrep/det398 24249743 10.1093/humrep/det398

