# Supplementary material for: Granulocyte colony‐stimulating factor induced T‐cell hyporesponsiveness via modulation of CD177+S100Ahi neutrophils in unexplained recurrent pregnancy loss
Source: Clin Transl Med. 2025 Oct 16;15(10):e70508. doi: 10.1002/ctm2.70508 (PMC12531352; doi:10.1002/ctm2.70508)
Supplement: Supplementary file 1 — Supporting Information [file CTM2-15-e70508-s001.docx]

**SUPPLEMENTAL INFORMATION**

**Granulocyte colony-stimulating factor induced T cell hyporesponsiveness via modulation of CD177^+^S100A^hi^ neutrophils in unexplained recurrent pregnancy loss**

Ping-Fen Li, M.D.^1^, Xue Zhang, M.D.^3^, Peng-Sheng Zheng, M.D.; Ph.D.^1, 2*^

# Supplementary Materials. This includes the supplemental methods, nine supplemental figures, and six supplemental tables.

## Supplemental Methods

### Patient characteristics

The selection criteria for patients with URPL were: two or more sequential spontaneous miscarriages with the same sexual partner before 28 weeks of gestation; 35 years ≥ age ≥ 20 years; natural pregnancy; couples with normal karyotypes and normal karyotype of embryonic tissues in the last miscarriage; and no other known risk factors, such as anatomical factors (congenital or acquired uterine malformation), autoimmune disorders, metabolic diseases, thrombophilia, obesity (BMI >30 kg/m^2^), or lifestyle factors (stress, smoking and excessive alcohol consumption). The first trimester of pregnancy was confirmed by ultrasound measurement of the crown-rump diameter. The basic clinical and laboratory characteristics are described in Table. S1-2.

### Administration of G-CSF

In total, 20 patients with URPL at 28–40 days of gestation were treated with recombinant G-CSF (150 µg subcutaneously daily) until the end of the ninth week. If the white blood cell counts exceeded 30×10^9^ /L during medication, G-CSF was temporarily discontinued and resumed after the count was reduced during reexamination.

### scRNA-seq data analysis

Raw reads were processed to generate gene expression profiles using CeleScope v1.5.2 (Singleron Biotechnologies) with default parameters. The Cell Ranger pipeline was used to align the scRNA-seq data with the human reference genome GRCh38 (hg38). Only cells with gene counts between 200 and 5,000, unique molecular identifier (UMI) counts more than top 2%, and mitochondrial content below 10% were retained for quality control. Doublets were discarded using the DoubletFinder R package. The data for each sample were normalized and scaled using the NormalizeData and ScaleData functions in Seurat (v.3.1.1). Principal component analysis was conducted on the top 2000 features with high inter-cell variation using the FindVariableFeatures function, and Harmony v1.0 was used to eliminate batch effects between the samples. The data were subsequently subjected to dimensionality reduction and cluster analysis following the workflow described by the Seurat software package, with a cell cluster resolution parameter of 0.5. Each cluster was annotated using the SynEcoSys database, integrated with the expression patterns of canonical markers, and visualized using the RunUMAP function. To identify DEGs, we used the scanpy.tl.rank_genes_groups function based on the Wilcoxon rank sum test with default parameters. Additionally, we selected the genes expressed in more than 10% of the cells in either of the compared groups of cells, with an average log (Fold Change) value greater than 1.25, as DEGs. Enrichment analysis was performed using the “clusterProfiler” R package v 3.16.1. UCell scores in the T/NK lineage were based on the Mann–Whitney U statistic by ranking query genes in the order of their expression levels in individual cells.

To explore the differentiation trajectories of PB neutrophils under G-CSF stimulation, we used Monocle v2 to perform a pseudotime analysis. The top 1000 highly variable genes (mean expression ≥ 0.1 and empirical dispersion ≥ 2× fitted dispersion) were selected from neutrophil subclusters to order cells, and CytoTRACE was used to predict the differentiation status of each neutrophil subcluster. We then visualized the developmental trajectories and dynamic changes in gene expression along with pseudotime using TradeSeq (v1.6.0). The SCENIC algorithm was applied to evaluate the transcription factors and related regulatory networks in each neutrophil subcluster.

Systematic communication analysis among the nine clusters was conducted by CellPhone DB and CellChat. We identified the null distribution of the average ligand–receptor pair expressed in neutrophils and T cells before and after drug treatment following the default settings. Ligand–receptor interaction pairs with p <0.05 and an average log expression >0.1 were considered significant. Downstream target genes of the predicted ligand receptors, coupled with major signaling inputs and outputs, were used to further explore the regulatory effects of differential communication on T cells.

### RNA extraction and quantitative real-time PCR (qRT-PCR)

Total RNA was extracted from magnetic bead-sorted neutrophils (at least 10^6^) using a SteadyPure RNA Extraction Kit (AG, China) according to the manufacturer's instructions. cDNA was synthesized from 1 μg RNA templates using the Evo M-MLV RT Mix Kit (AG, China). qRT-PCR was performed using SYBR Green Pro Taq (AG, China) and gene-specific primer pairs (Table. S5) using a qRT-PCR instrument (Gentier 96E/96R, TIANLONG). All samples were detected in triplicates; the relative gene expression was analyzed using the 2-^ΔΔCT^ method with normalization to 18srRNA.

### Preparation of single cell suspension of decidual tissue

Decidual tissues were thoroughly rinsed with sterile phosphate-buffered saline and mechanically minced into 1–2 mm³ fragments. The fragments were enzymatically dissociated by incubation with a 10× volume of Accutase® solution (Invitrogen, USA) at 37°C for 1 hour. The obtained cell suspension was sequentially filtered through 70μm and 40μm sterile nylon mesh to remove residual tissue debris. Filtered cells were resuspended in RPMI 1640 medium supplemented with 10% FBS and cultured in adherent plates for 2 hours at 37°C to facilitate stromal component depletion. Non-adherent cells were collected and further purified via density gradient centrifugation for neutrophils enrichment.

### HE Staining

Deparaffinization and hydration procedures are the same as those in the immunohistochemistry protocols. Nuclei are stained with hematoxylin for 5 min, followed by differentiation in 1% hydrochloric acid-ethanol solution. Cytoplasmic counterstaining employs eosin dye for 1-3 minutes. Sequential dehydration through graded ethanol series precedes xylene-based clearing. Permanent mounting is achieved using synthetic resin medium.

**SUPPLEMENTARY FIGURES**


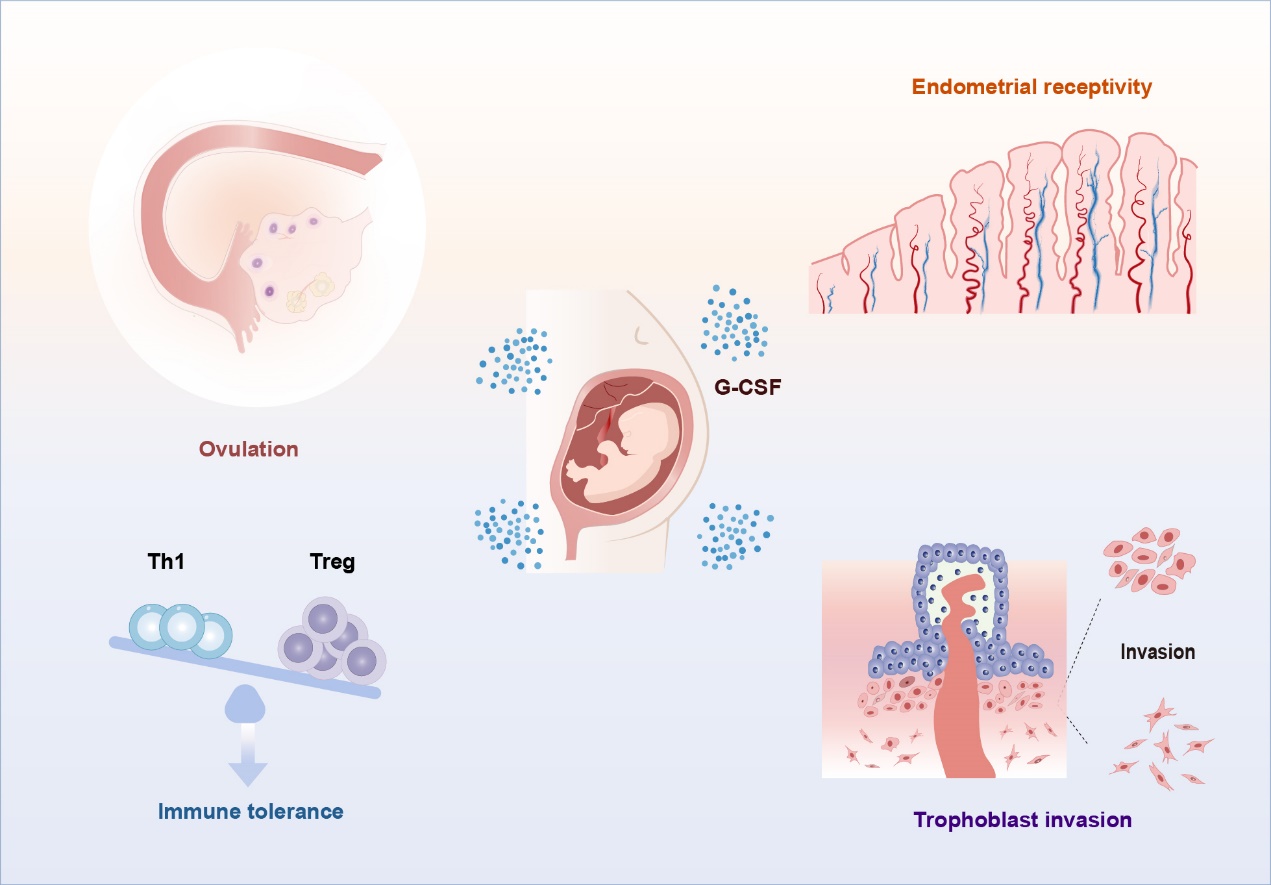


**Fig. S1. Function of G-CSF in reproductive field.**

**
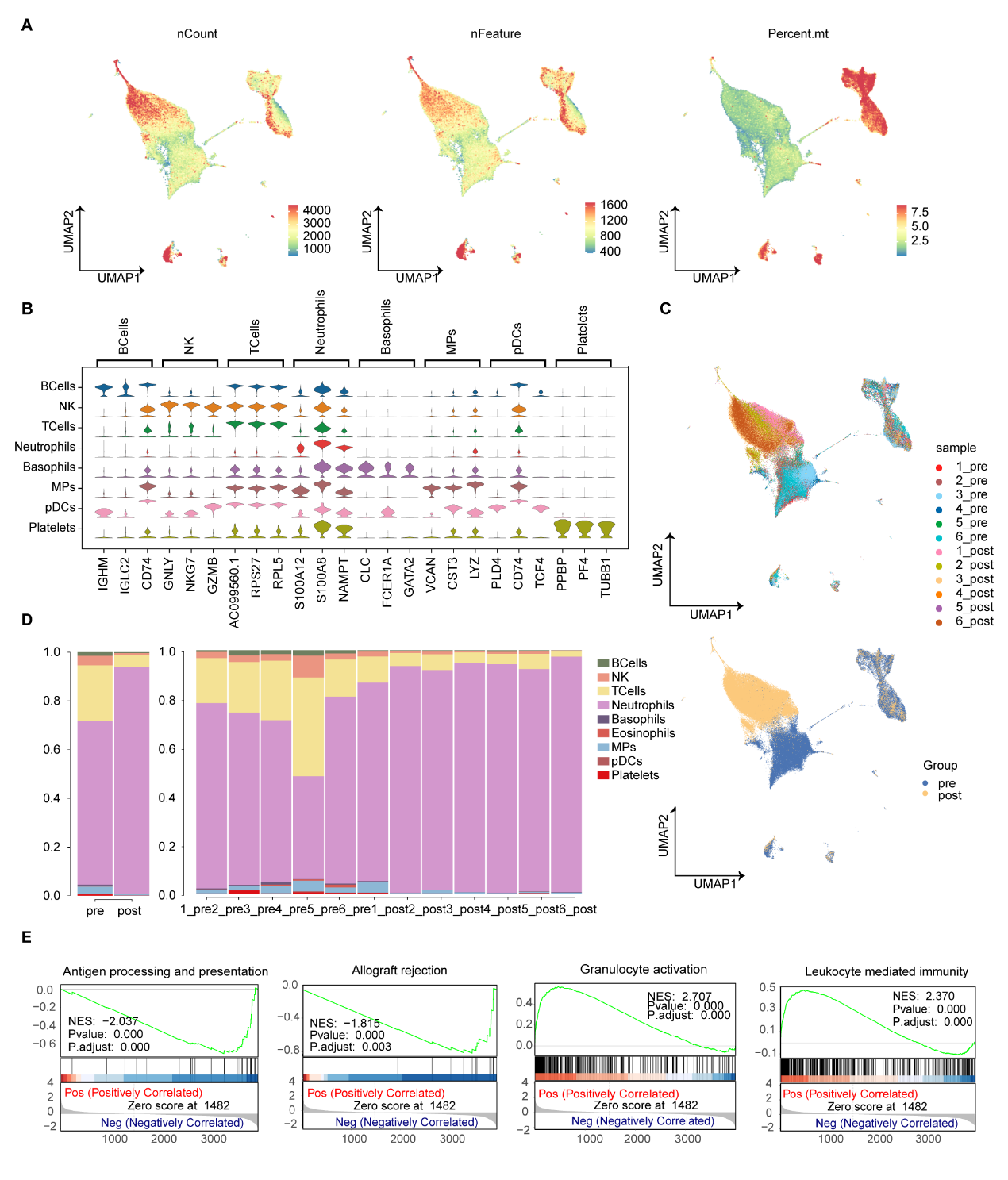
Fig. S2. Overview of quality and characteristics of clustering cells in G-PB.**

**(A)** UMAP showing UMIs (left), gene counts (middle) and percentage of mitochondrial genes (right) for all samples.

**(B)** Violin plot showing expression distribution of top 3 DEGs in each lineage.

**(C)** UMAP visualization of PB cells from 6 donors before and after G-CSF treatment. Colored according to samples (top) and sample stage (bottom).

**(D)** Bar plot displaying the alterations in the proportion of PB cell clusters across 6 samples before and after G-CSF treatment.

**(E)** Enrichment of GSEA terms for the DEGs in G-PB compared with unstimulated PB.

UMAP, uniform manifold approximation and projection; UMI, unique molecular identifier; PB, peripheral blood; DEGs, differentially expressed genes; G-PB, G-CSF-mobilized peripheral blood; GSEA, Gene Set Enrichment Analysis.


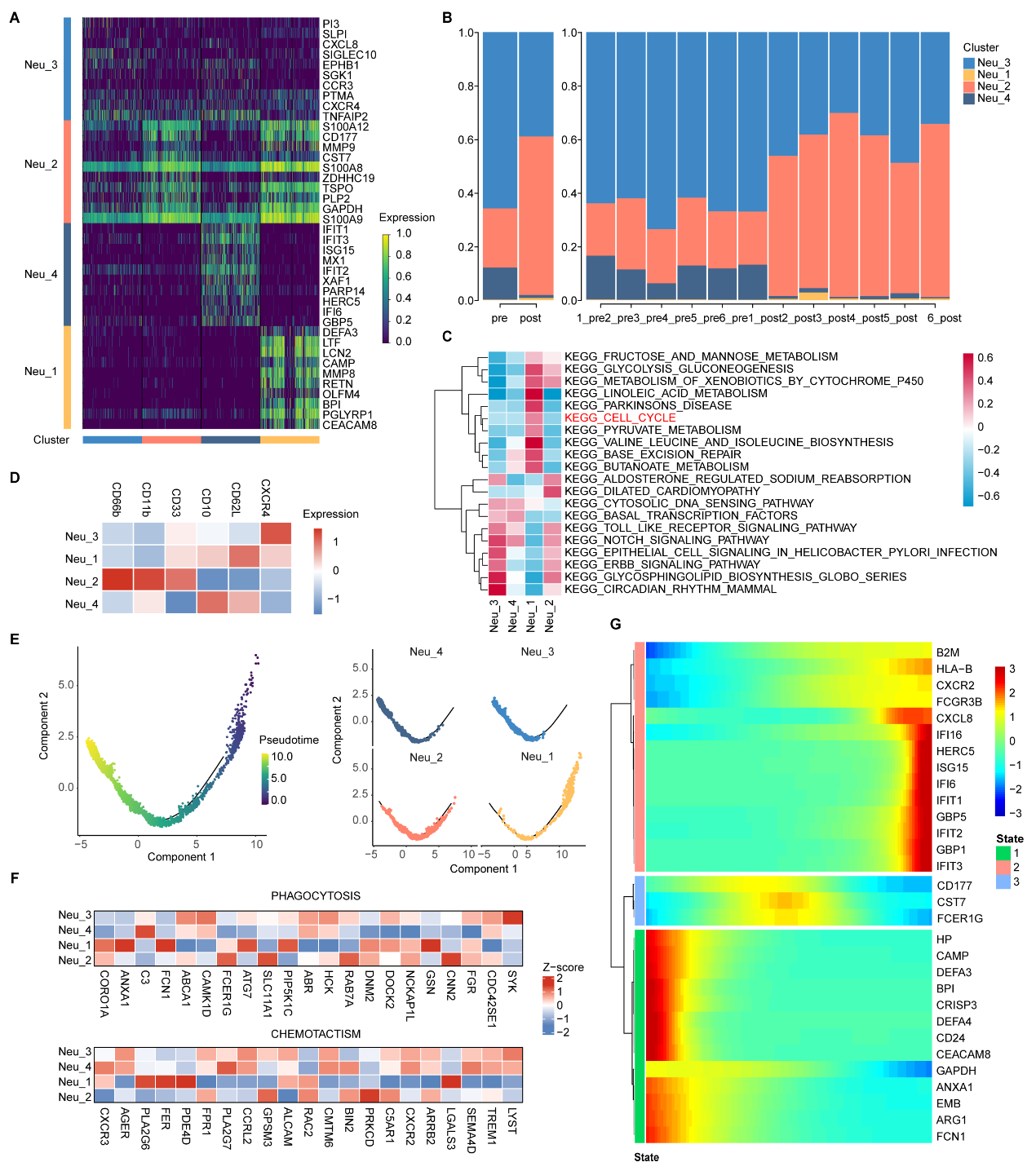


**Fig. S3. Heterogeneous neutrophil differentiation and development upon G-CSF stimulation.**

**(A)** Heatmap displaying scaled expression of the top10 DEGs across 4 neutrophil subclusters.

**(B)** Bar plot displaying the alterations in the proportion of neutrophil subclusters across 6 samples before and after G-CSF treatment.

**(C)** Heatmap of differential pathways according to GSVA scores for 4 neutrophil subclusters.

**(D)** Heatmap displaying scaled expression of classic surface marker genes in 4 neutrophil subclusters.

**(E)** Pseudo-time ordering of the development of 4 neutrophil subclusters.

**(F)** Heatmap displaying scaled expression of phagocytosis- (F, top) and chemotaxis-critical (F, bottom) genes in 4 neutrophil subclusters.

**(G)** Heatmap hierarchical clustering displaying the dynamic changes of gene expression along with pseudo-time.

GO, Gene Ontology; DEGs, differentially expressed genes; GSVA, Gene Set Variation Analysis


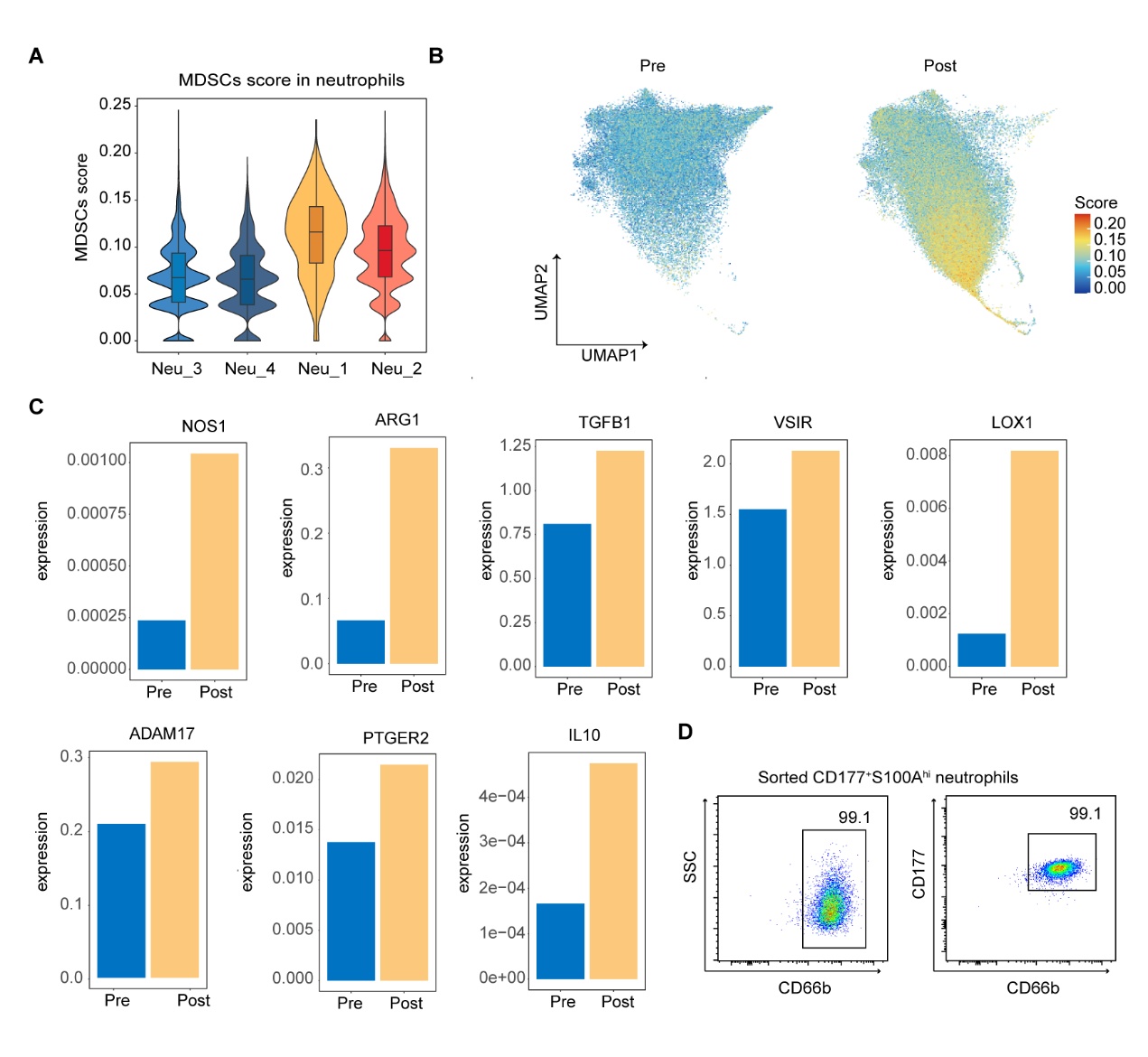


**Fig. S4. Immunomodulatory characteristics of neutrophils after G-CSF treatment.**

**(A)** Violin plots showing MDSCs scores across 4 neutrophil subclusters.

**(B)** UMAP showing MDSCs scores of 4 neutrophils between pre and post group. Pre represents samples before G-CSF treatment and post represents samples after G-CSF treatment.

**(C)** Box plot showing average expression of factors mediating the immunosuppressive properties of MDSCs before and after G-CSF treatment.

**(D)** Representative plots of purity of sorted CD177^+^S100A^hi^ neutrophils.

MDSCs, myeloid-derived suppressor cells


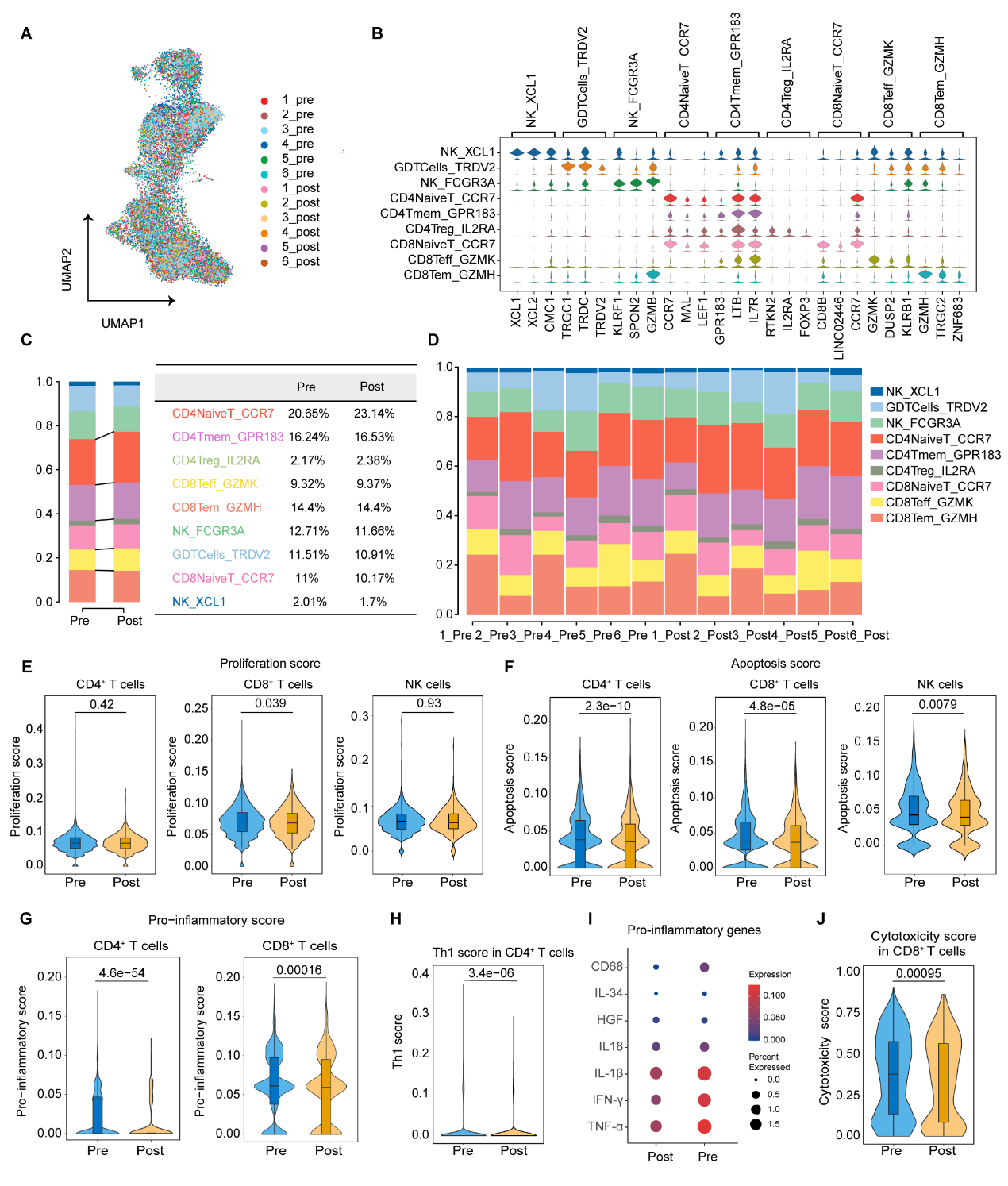


**Fig. S5. The impacts of G-CSF on T and NK lineage in PB.**

**(A)** UMAP of T and NK cells in 6 samples before and after G-CSF administration. Colored by samples.

**(B)** Violin plot displaying scaled expression of top 3 DEGs in T and NK cells.

**(C-D)** Proportion of T and NK subclusters in G-CSF mobilized PB compared with unstimulated PB (C) as well as each sample (D).

**(E-F)** Violin plot showing proliferation score (E) and apoptosis score (F) in CD4^+^ T cells (left), CD8^+^ T cells (middle) and NK cells(right) between unstimulated PB and G-PB.

**(G)**Violin plot showing pro-inflammatory score in CD4^+^ T cells (left) and CD8^+^ T cells (right) between unstimulated PB and G-PB.

**(H)** Violin plot showing Th1 score in CD4^+^ T cells between unstimulated PB and G-PB.

**(I)** Dot plot showing average expression of genes related to pro-inflammation before and after G-CSF treatment. The shadings and size of the circle denote the levels of scaled expression and the percentage of expressed cells.

**(J)** Violin plot showing cytotoxicity score in CD8^+^ T cells between unstimulated PB and G-PB.

UMAP, uniform manifold approximation and projection; PB, peripheral blood; G-PB, G-CSF-mobilized peripheral blood; DEGs, differentially expressed genes.


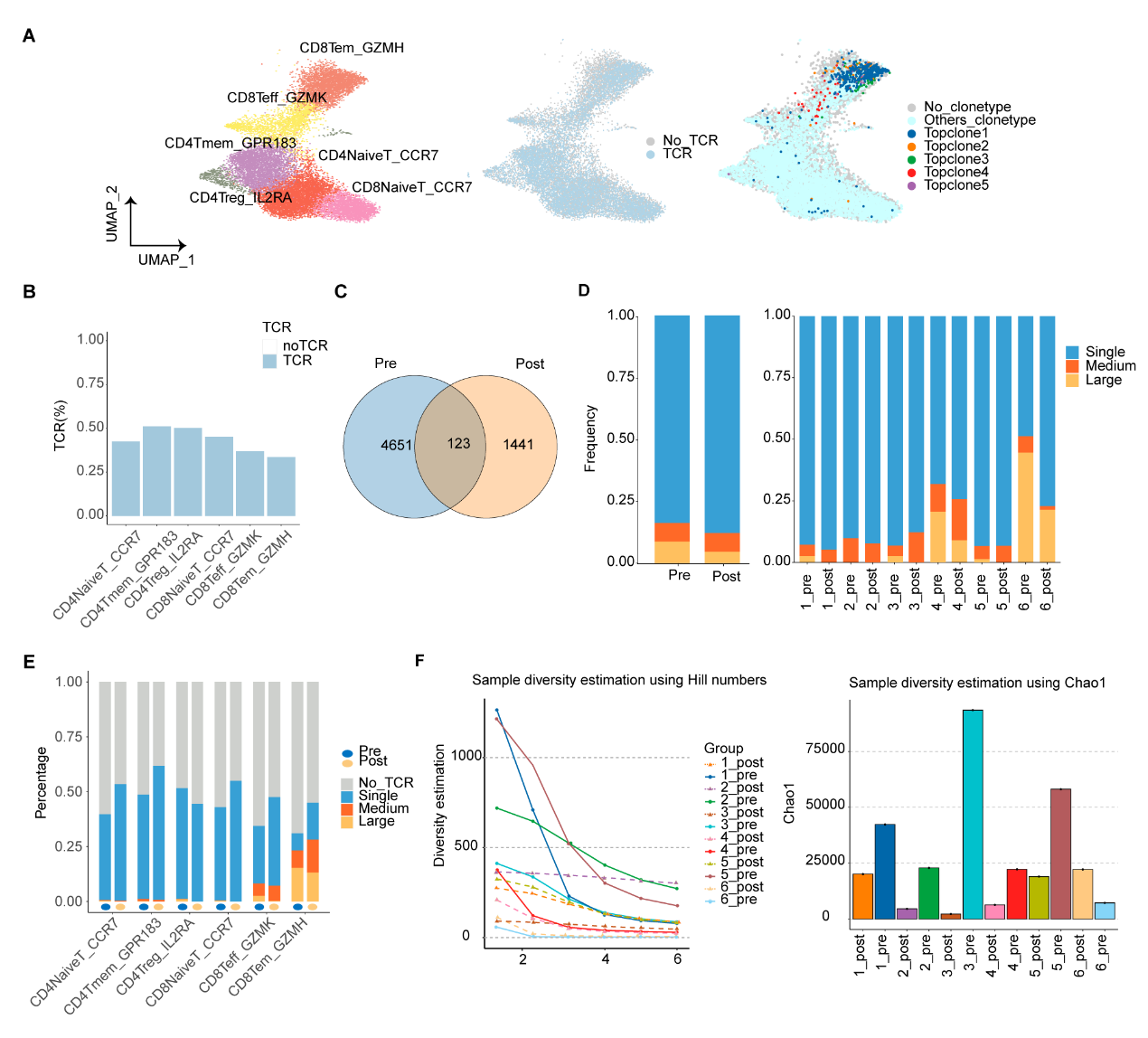


**Fig. S6. The impacts of G-CSF on TCR clonotypes.**

**(A)** UMAP representing identified subclusters (left), TCR detection (middle) and distribution of top 5 clonotypes (right) in T cells.

**(B)** Bar plot showing the proportion of TCR detected in each T subcluster.

**(C)** Venn plot showing total number of clonotypes in unstimulated PB compared with G-PB.

**(D)** Bar plot of proportion of different levels of clonotype across 6 sample before and after G-CSF treatment. Pre indicates samples before G-CSF treatment and post represents samples after G-CSF treatment.

**(E)** Bar plot of proportion of different levels of clonotype in each subcluster before and after G-CSF treatment.

**(F)** Clonal diversity inferred by Hill (left) and Chao1 (right) indices across six samples before and after G-CSF administration. Solid lines with circles (●) indicate pre-treatment samples; dashed lines with triangles (▲) represent post-treatment samples.

UMAP, uniform manifold approximation and projection; TCR, T cell receptor; PB, peripheral blood.


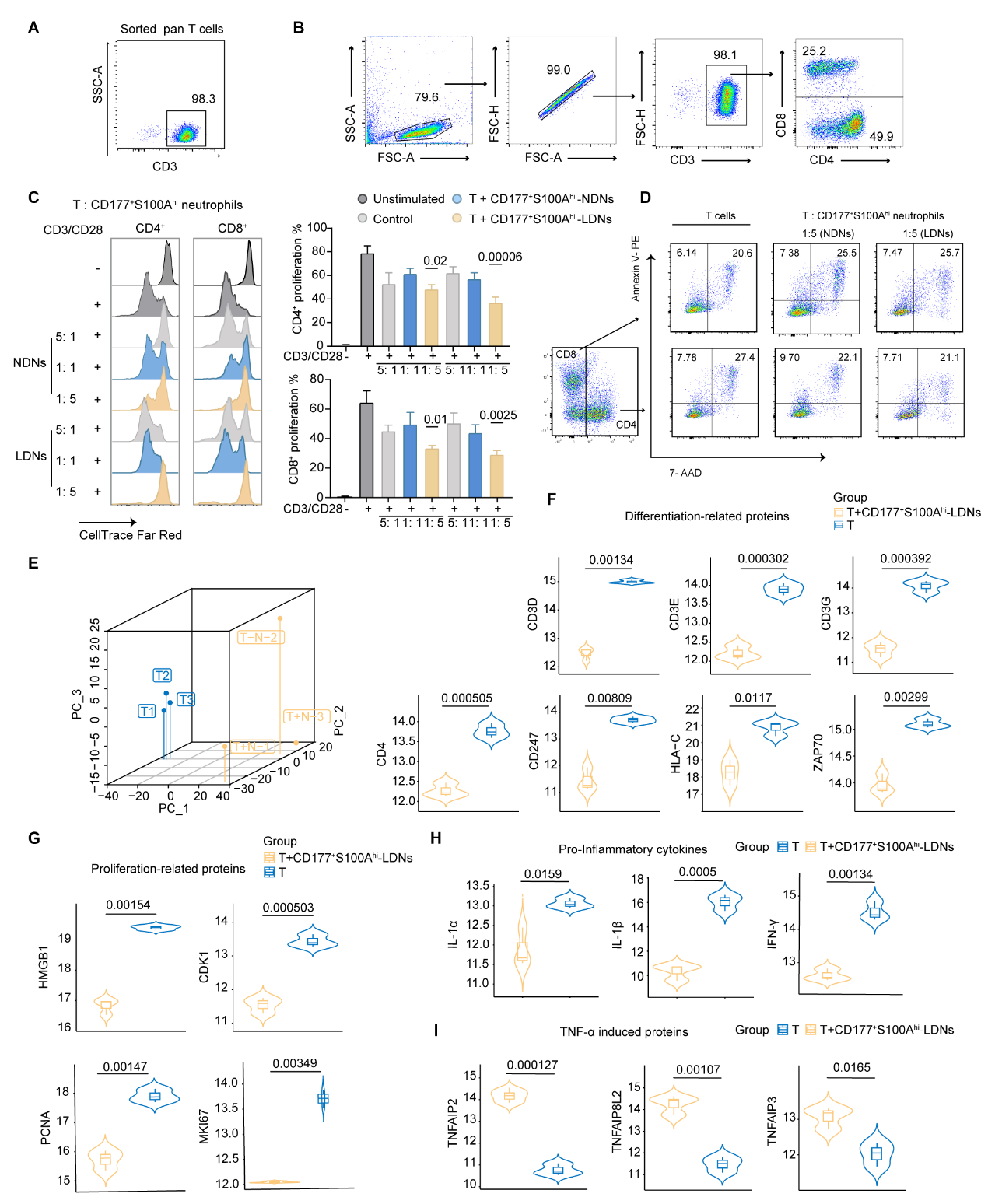


**Fig. S7. Immunosuppressive capacity of circulating CD177^+^S100A^hi^ neutrophils.**

1. Representative plots of purity of sorted pan-T cells.

**(B)** Flow cytometry showing the gating strategy for T cells (gated on CD3^+^CD4^+^/CD3^+^CD8^+^).

**(C)** Representative images of proliferative alterations (left), proportion of proliferative CD4^+^ T cells (right top) and CD8^+^ T cells (right bottom) in cocultures of pan-T cells with or without CD177^+^S100A^hi^-LDNs and CD177^+^S100A^hi^-NDNs (ratios of 5:1, 1:1, 1:5) at day 3.5 (n = 5). Data are represented as mean ± SEM.

**(D)** Representative images of apoptosis of CD4^+^ T cells and CD8^+^ T cells at a coculture ratio of 1:5.

**(E)** PCA cluster of T cells exposed to CD177^+^S100A^hi^-LDNs and cultured alone.

**(F-I)** Violin plot of average expression of T cell differentiation-related proteins (F), proliferation-related proteins (G), pro-inflammatory cytokines (H), and TNF-α induced proteins (I) in T cells exposed to CD177^+^S100A^hi^-LDNs compared with cultured alone. The Y-axis represents the value of the log2 protein abundance.

*p* values were calculated using the one-way ANOVA with Tukeys post-hoc test and Student’s t-test.

LDNs, low density neutrophils; NDNs, normal density neutrophils; TNF-α, tumor necrosis factor-α; IFN-γ, interferon-γ; PCA, principal component analysis.


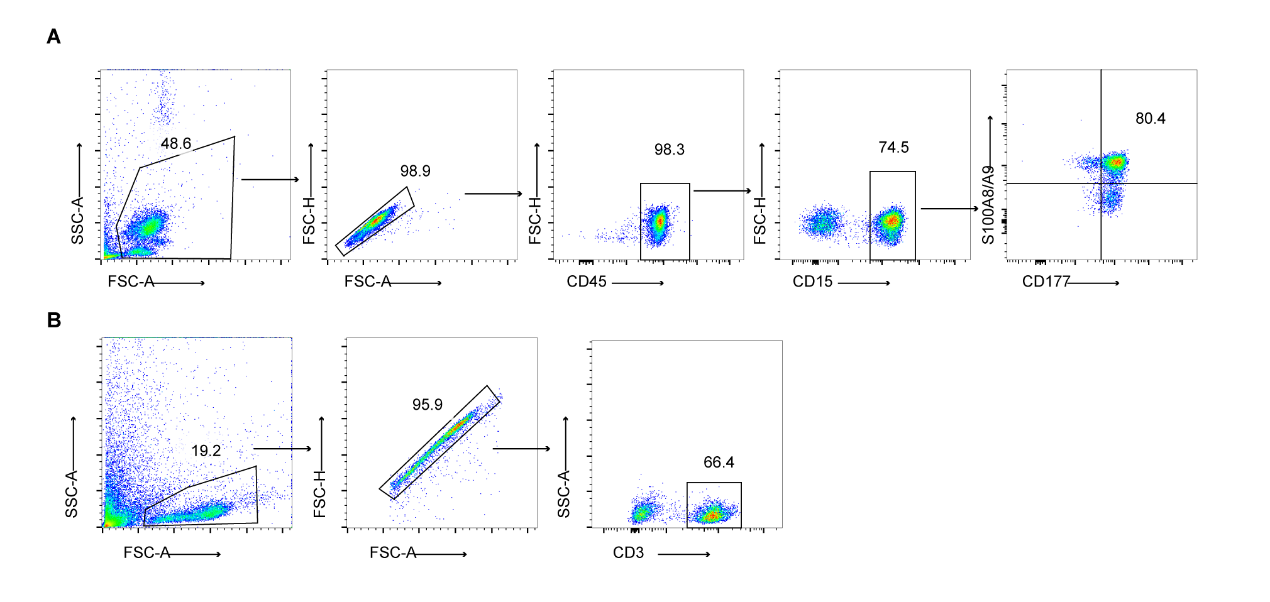


**Fig. S8. Flow cytometry gating strategy of CD177^+^S100A^hi^ neutrophils and CD3⁺ T cells in peripheral blood.**

**(A)** Flow cytometry showing the gating strategy for identifying CD177⁺S100Aʰⁱ neutrophils (gated on CD45^+^CD15^+^CD177^+^S100A^hi^).

**(B)** Flow cytometry showing the gating strategy for quantifying CD3⁺ T cells frequencies within PBMCs.

PBMCs, Peripheral blood mononuclear cells.


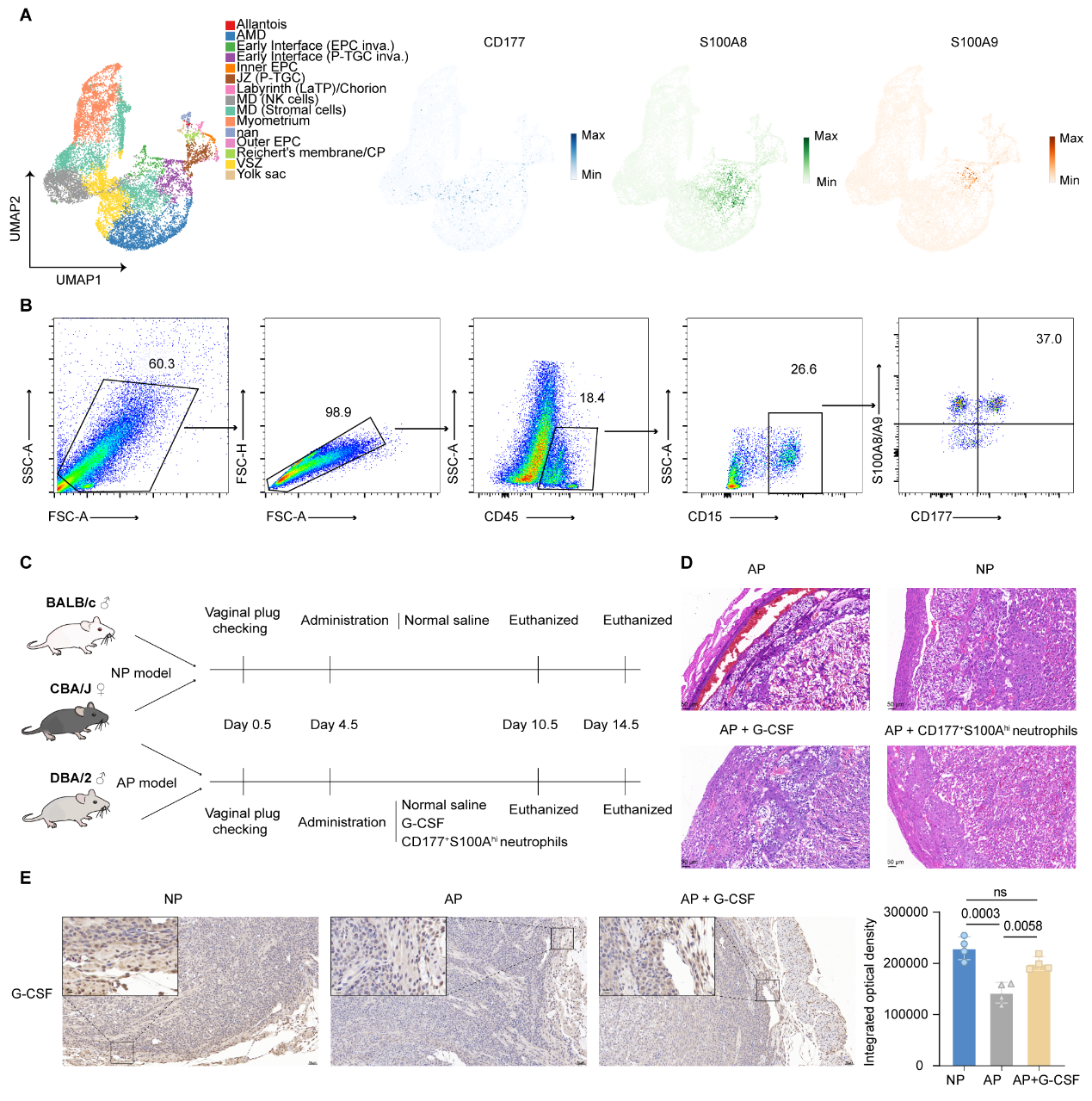


**Fig. S9. The protective role of CD177^+^S100A^hi^ neutrophils during pregnancy.**

**(A)** UMAP of CD177, S100A8, and S100A9 expression at the maternal-fetal interface at E7.5

**(B)** Flow cytometry showing the gating strategy for identifying CD177⁺S100Aʰⁱ neutrophils in decidua (gated on CD45^+^CD15^+^CD177^+^S100A^hi^).

**(C)** Schematic representation of animal experimental protocol. CBA/J females were mated with BALB/c males and DBA/2 males to establish NP and AP models, respectively.

**(D)** H&E staining of midsagittal sections of the placenta in NP, AP, AP model treated with G-CSF or CD177^+^S100A^hi^ neutrophils. Scale bar, 50μm.

**(E)** Representative images (left) and quantitative analysis (right) of G-CSF expression in NP, AP and AP model treated with G-CSF (each group: n = 4). Scale bar, 50μm, 10μm. Data are represented as mean ± SD.

Significances were calculated by the one-way ANOVA with Tukeys post-hoc test.

HCs, healthy pregnant controls; URPL, unexplained recurrent pregnancy loss; AP, abortion-prone; NP, normal pregnancy.

**SUPPLEMENTARY TABLES**

**Table. S1**

**Characteristics of PB samples of HCs, patients with URPL and URPL receiving G-CSF treatment**

|  | HCs (n=13) | URPL (n=18) | URPL+G-CSF (n=20) |
| --- | --- | --- | --- |
| Age (years) | 29.77 ± 1.92 | 31.25 ± 2.27 | 31.55 ± 2.35 |
| BMI (kg/m²) | 20.81 ± 1.49 | 21.76 ± 2.49 | 21.78 ± 2.25 |
| Miscarriage < 28 weeks | 0.00 ± 0.00 | 2.75 ± 085 | 2.85 ± 1.09 |
| Menstrual cycle (days) | 30.62 ± 2.60 | 31.38 ± 3.01 | 31.15 ± 3.01 |
| APS/ ANA/ RF antibodies | - | - | - |

HCs, healthy pregnancy controls; URPL, unexplained recurrent pregnancy loss; BMI: body mass index; APS, antiphospholipid syndrome; ANA, antinuclear antibody; RF, rheumatoid factor.

Data are represented as mean ± SD.

**Table. S2**

**Characteristics of decidual samples of first trimester from HCs, women with a history of URPL and women with URPL receiving G-CSF treatment**

|  | URPL (n=8) | HC (n=8) | URPL + G-CSF (n=6) |
| --- | --- | --- | --- |
| Age(years) | 31.38 ± 3.38 | 27.50 ± 5.01 | 31.17 ± 3.19 |
| Gestation age (days) | 58.88 ± 5.33 | 52.50 ± 8.31 | 57.67 ± 7.58 |
| BMI (kg/m²) | 21.34 ± 1.91 | 20.65 ± 2.09 | 21.40 ± 2.08 |
| Miscarriage < 28weeks | 2.38 ± 0.74 | 0.38 ± 0.52 | 2.17 ± 0.41 |
| Menstrual cycle (days) | 31.00 ± 2.67 | 30.75 ± 3.15 | 30.50 ± 3.21 |
| APS/ ANA/ RF antibodies | - | - | - |

HCs, healthy pregnancy controls; URPL, unexplained recurrent pregnancy loss; BMI: body mass index; APS, antiphospholipid syndrome; ANA, antinuclear antibody; RF, rheumatoid factor.

Data are represented as mean ± SD.

**Table. S3**

**Total number of cells that met the requirement of quality control and subjected to single-cell sequencing**

| Group | ID | Blood(obtained) | Blood(retained) |
| --- | --- | --- | --- |
| Pre | case1 | 10,113 | 9472 |
|  | case2 | 12,489 | 11765 |
|  | case3 | 10,750 | 9949 |
|  | case4 | 17,646 | 15831 |
|  | case5 | 10,235 | 9681 |
|  | case6 | 13,860 | 13033 |
| Post | case7 | 14,357 | 13753 |
|  | case8 | 10,922 | 10563 |
|  | case9 | 16,790 | 16124 |
|  | case10 | 14,403 | 13973 |
|  | case11 | 11,822 | 11457 |
|  | case12 | 10,769 | 10418 |

**Table. S4**

The clonal status of each T cell across 12 samples

| sample | celltype | Large | Medium | Single |
| --- | --- | --- | --- | --- |
| 1_post | CD4NaiveT_CCR7 | 0 | 2 | 102 |
| 1_post | CD4Tmem_GPR183 | 0 | 0 | 89 |
| 1_post | CD4Treg_IL2RA | 0 | 0 | 9 |
| 1_post | CD8NaiveT_CCR7 | 0 | 0 | 45 |
| 1_post | CD8Teff_GZMK | 0 | 2 | 29 |
| 1_post | CD8Tem_GZMH | 0 | 12 | 8 |
| 1_pre | CD4NaiveT_CCR7 | 0 | 4 | 409 |
| 1_pre | CD4Tmem_GPR183 | 0 | 16 | 463 |
| 1_pre | CD4Treg_IL2RA | 0 | 1 | 66 |
| 1_pre | CD8NaiveT_CCR7 | 0 | 1 | 279 |
| 1_pre | CD8Teff_GZMK | 2 | 25 | 96 |
| 1_pre | CD8Tem_GZMH | 36 | 17 | 30 |
| 2_post | CD4NaiveT_CCR7 | 0 | 0 | 99 |
| 2_post | CD4Tmem_GPR183 | 0 | 2 | 107 |
| 2_post | CD4Treg_IL2RA | 0 | 0 | 15 |
| 2_post | CD8NaiveT_CCR7 | 0 | 0 | 53 |
| 2_post | CD8Teff_GZMK | 0 | 13 | 54 |
| 2_post | CD8Tem_GZMH | 0 | 15 | 27 |
| 2_pre | CD4NaiveT_CCR7 | 0 | 4 | 210 |
| 2_pre | CD4Tmem_GPR183 | 0 | 0 | 236 |
| 2_pre | CD4Treg_IL2RA | 0 | 0 | 36 |
| 2_pre | CD8NaiveT_CCR7 | 0 | 0 | 85 |
| 2_pre | CD8Teff_GZMK | 0 | 31 | 110 |
| 2_pre | CD8Tem_GZMH | 0 | 41 | 52 |
| 3_post | CD4NaiveT_CCR7 | 0 | 0 | 28 |
| 3_post | CD4Tmem_GPR183 | 0 | 1 | 31 |
| 3_post | CD4Treg_IL2RA | 0 | 0 | 2 |
| 3_post | CD8NaiveT_CCR7 | 0 | 0 | 13 |
| 3_post | CD8Teff_GZMK | 0 | 4 | 10 |
| 3_post | CD8Tem_GZMH | 0 | 6 | 8 |
| 3_pre | CD4NaiveT_CCR7 | 0 | 0 | 135 |
| 3_pre | CD4Tmem_GPR183 | 0 | 0 | 137 |
| 3_pre | CD4Treg_IL2RA | 0 | 0 | 19 |
| 3_pre | CD8NaiveT_CCR7 | 0 | 0 | 78 |
| 3_pre | CD8Teff_GZMK | 1 | 3 | 39 |
| 3_pre | CD8Tem_GZMH | 10 | 13 | 24 |
| 4_post | CD4NaiveT_CCR7 | 0 | 0 | 74 |
| 4_post | CD4Tmem_GPR183 | 0 | 0 | 49 |
| 4_post | CD4Treg_IL2RA | 0 | 0 | 6 |
| 4_post | CD8NaiveT_CCR7 | 0 | 0 | 56 |
| 4_post | CD8Teff_GZMK | 0 | 7 | 29 |
| 4_post | CD8Tem_GZMH | 22 | 37 | 32 |
| 4_pre | CD4NaiveT_CCR7 | 1 | 0 | 166 |
| 4_pre | CD4Tmem_GPR183 | 0 | 2 | 138 |
| 4_pre | CD4Treg_IL2RA | 1 | 0 | 16 |
| 4_pre | CD8NaiveT_CCR7 | 2 | 2 | 129 |
| 4_pre | CD8Teff_GZMK | 32 | 17 | 50 |
| 4_pre | CD8Tem_GZMH | 75 | 77 | 52 |
| 5_post | CD4NaiveT_CCR7 | 0 | 1 | 124 |
| 5_post | CD4Tmem_GPR183 | 0 | 2 | 95 |
| 5_post | CD4Treg_IL2RA | 0 | 0 | 7 |
| 5_post | CD8NaiveT_CCR7 | 0 | 0 | 61 |
| 5_post | CD8Teff_GZMK | 0 | 4 | 35 |
| 5_post | CD8Tem_GZMH | 0 | 18 | 13 |
| 5_pre | CD4NaiveT_CCR7 | 0 | 2 | 443 |
| 5_pre | CD4Tmem_GPR183 | 0 | 7 | 359 |
| 5_pre | CD4Treg_IL2RA | 1 | 0 | 45 |
| 5_pre | CD8NaiveT_CCR7 | 0 | 1 | 276 |
| 5_pre | CD8Teff_GZMK | 0 | 17 | 108 |
| 5_pre | CD8Tem_GZMH | 17 | 43 | 31 |
| 6_post | CD4NaiveT_CCR7 | 0 | 0 | 93 |
| 6_post | CD4Tmem_GPR183 | 0 | 0 | 56 |
| 6_post | CD4Treg_IL2RA | 0 | 0 | 6 |
| 6_post | CD8NaiveT_CCR7 | 0 | 0 | 26 |
| 6_post | CD8Teff_GZMK | 1 | 0 | 17 |
| 6_post | CD8Tem_GZMH | 57 | 2 | 12 |
| 6_pre | CD4NaiveT_CCR7 | 11 | 4 | 139 |
| 6_pre | CD4Tmem_GPR183 | 5 | 3 | 108 |
| 6_pre | CD4Treg_IL2RA | 2 | 0 | 22 |
| 6_pre | CD8NaiveT_CCR7 | 3 | 0 | 25 |
| 6_pre | CD8Teff_GZMK | 9 | 4 | 53 |
| 6_pre | CD8Tem_GZMH | 272 | 22 | 22 |

**Table. S5**

**Primers used for quantitative real-time PCR**

| Genes | Primers |
| --- | --- |
| CD177 forward | CTGGTTCACGTCTCCAAACC |
| CD177 reverse | TCTCCTGCAGTTGCTCAGAT |
| S100A8 forward | GGGATGACCTGAAGAAATTGCTA |
| S100A8 reverse | TGTTGATATCCAACTCTTTGAACC |
| S100A9 forward | GTGCGAAAAGATCTGCAAAATTT |
| S100A9 reverse | [GGTCCTCCATGATGTGTTCTATGA](https://www.sciencedirect.com/topics/biochemistry-genetics-and-molecular-biology/glucose-test) |
| S100A12 forward | CTGTGCATTGAGGGGTTAACATTAG |
| S100A12 reverse | CCGAACTGAGTATTGGTGGAAGAT |
| 18S rRNA forward | GTAACCCGTTGAACCCCATT |
| 18S rRNA reverse | CCATCCAATCGGTAGTAGCG |

**Table. S6**

**Experimental resources used in the study**

| **REAGENTS** | **SOURCE** | **IDENTIFIER** |
| --- | --- | --- |
| **Antibodies** |  |  |
| Purified NA/LE Human BD Fc Block™ | BD bioscience | Cat# 564765 |
| PerCP-Cy™5.5 Mouse Anti-Human CD3 | BD bioscience | Cat# 560835 |
| APC-Cy™7 Mouse Anti-Human CD4 | BD bioscience | Cat# 557871 |
| APC Mouse Anti-Human CD3 | BD bioscience | Cat# 555342 |
| Alexa Fluor® 647 Mouse Anti-Human S100A8/A9 | BD bioscience | Cat# 566010 |
| Alexa Fluor® 647 Rat Anti-Mouse CD177 | BD bioscience | Cat# 566599 |
| PE-Cy™7 Rat Anti-Mouse Ly-6G | BD bioscience | Cat# 560601 |
| PerCP/cyanine5.5 anti-human CD15 | Biolegend | Cat# 301921 |
| APC anti-human CD15 | Biolegend | Cat# 301907 |
| APC anti-human Arginase I Antibody | Biolegend | Cat# 369705 |
| FITC anti-human CD177 Antibody | Biolegend | Cat# 315803 |
| PE anti-human Arginase I Antibody | Biolegend | Cat# 369703 |
| CD8a Monoclonal Antibody (RPA-T8), PE-Cyanine7 | Thermo | Cat# 25-0088-41 |
| CD177 Rabbit mAb | Abclonal | Cat# A24263 |
| CSF3 Rabbit pAb | Abclonal | Cat# A6178 |
| S100A9 Rabbit PolymAb | Abclonal | Cat# A26782PM |
| ABflo® 488 Rabbit anti-Mouse S100A9 mAb | Abclonal | A26915 |
| CD177 Antibody, anti-human, FITC, REAfinity | Miltenyi | Cat#130-126-380 |
| CD177 Polyclonal Antibody | Huaxingbio | Cat# HX13023 |
| **Chemicals, buffers and recombinant proteins** |  |  |
| 7AAD | BD bioscience | Cat# 559925 |
| Stain Buffer (FBS) | BD bioscience | Cat# 554656 |
| Perm/Wash™ Perm/Wash Buffer | BD bioscience | Cat# 554723 |
| Propidium Iodide Staining Solution | BD bioscience | Cat# 556463 |
| MACS® BSA Stock Solution | Miltenyi | Cat# 130-091-376 |
| autoMACS® Rinsing Solution | Miltenyi | Cat# 130-091-222 |
| CD15 MicroBeads, human | Miltenyi | Cat# 130-046-601 |
| Anti-FITC MicroBeads | Miltenyi | Cat# 130-048-701 |
| Recombinant Human IL-2 Protein | Abclonal | Cat# RP01039 |
| N -Acetyl-L-cysteine | Sigma | Cat# A9165 |
| Catalase | Sigma | Cat# C1345 |
| Accutase | Invitrogen | Cat# 00-4555-56 |
| L-Arginine | MCE | Cat# HY-N0455 |
| Granulocyte colony stimulating factor | Kexing | Cat# S20010016 |
| ImmunoCult™ Human CD3/CD28 T Cell Activator | Stemcell | Cat# 10971 |
| Lymphoprep™ | Stemcell | Cat# 07851 |
| Trypan Blue | Servicebio | Cat# G1019 |
| BioSample Stabilizing Reagent | AG | Cat# AG21015 |
| **Critical Commercial Assays （Kit and Consumables）** |  |  |
| Pan-T cell Isolation kit | Miltenyi | Cat# 130-096-535 |
| PE Annexin V Apoptosis Detection Kit I | BD bioscience | Cat# 559763 |
| Cytofix/Cytoperm™ Fixation/Permeabilization Kit | BD bioscience | Cat# 554714 |
| Treble-Fluorescence Immunohistochemistry Mouse/Rabbit Kit | Immunoway | Cat# RS0035 |
| Reactive Oxygen Species Assay Kit | Huaxingbio | Cat# HX3611-2 |
| Diff-Quick Stain Kit | TBD | Cat#TBD20180083 |
| Human peripheral blood neutrophil separation kit | TBD | Cat# LZS11131 |
| CellTrace™ Far Red Cell Proliferation Kit | Thermo | Cat# C34572 |
| Human IFN-gamma ELISA Kit | MultiSciences | Cat# EK180 |
| Human TNF-α High Sensitivity ELISA Kit | MultiSciences | Cat# EK182HS |
| Human L-Arg ELISA Kit | Coab Biotech | Cat# CB1061-Hu |
| SteadyPure Quick RNA Extraction Kit | AG | Cat# AG21023 |
| Evo M-MLV RT Mix Kit | AG | Cat# AG11728 |
| Multiple cytokine detection kit | Raisecare | Cat# R601001 |
| GEXSCOPE® Single Cell RNA Library Kit | Singleron |  |
| GEXSCOPE Single Cell Immuno-TCR Kit | Singleron |  |
| LS Columns | Miltenyi | Cat# 130-042-401 |
| SepMate™ | Stemcell | Cat# 86415 |
| HTS Transwell®-96 Permeable Support with 0.4 µm Pore Polycarbonate Membrane | Corning | Cat# 3391 |
| 96 well Culture Plate Round Bottom with lid | Corning | Cat# 3799 |
